# RACK1A positively regulates opening of the apical hook in *Arabidopsis thaliana* via suppression of its auxin response gradient

**DOI:** 10.1073/pnas.2407224122

**Published:** 2025-07-21

**Authors:** Qian Ma, Sijia Liu, Siamsa M. Doyle, Sara Raggi, Barbora Pařízková, Deepak Kumar Barange, Hemamshu Ratnakaram, Edward G. Wilkinson, Isidro Crespo Garcia, Joakim Bygdell, Gunnar Wingsle, Dirk Roeland Boer, Lucia C. Strader, Fredrik Almqvist, Ondřej Novák, Stéphanie Robert

**Affiliations:** ^a^Umeå Plant Science Centre, Department of Forest Genetics and Plant Physiology, Swedish University of Agricultural Sciences, Umeå SE-901 83, Sweden; ^b^Laboratory of Growth Regulators, Faculty of Science of Palacký University & Institute of Experimental Botany of the Czech Academy of Sciences, Olomouc CZ-783 71, Czech Republic; ^c^Department of Chemistry, Umeå University, Umeå SE-901 87, Sweden; ^d^Department of Biology, Duke University, Durham, NC 27008; ^e^BL13-XALOC Beamline, Experiments Division, ALBA Synchrotron, Cerdanyola del Vallès, Barcelona 08290, Spain; ^f^Computational Life Science Cluster, Department of Chemistry, Umeå University, Umeå SE-901 87, Sweden

**Keywords:** apical hook, auxin, *Arabidopsis*, differential cell growth

## Abstract

Apical hook development is an ideal model for studying differential growth in plants and is controlled by complex phytohormonal crosstalk, with auxin being the major player. Here, we identified a bioactive small molecule that decelerates apical hook opening in *Arabidopsis thaliana*. Our genetic studies suggest that this molecule enhances or maintains the auxin maximum found in the inner hook side and requires certain auxin signaling components to modulate apical hook opening. Using biochemical approaches, we then revealed the WD40 repeat scaffold protein RECEPTOR FOR ACTIVATED C KINASE 1A (RACK1A) as a direct target of this compound. We present data in support of RACK1A playing a positive role in apical hook opening by activating specific auxin signaling mechanisms and negatively regulating the differential auxin response gradient across the hook, thereby adjusting differential cell growth, an essential process for organ structure and function in plants.

## Significance

Differential growth, or the growth of cells at different rates across tissues, is essential for providing shape and structure during plant development. The apical hook is a transient structure formed by differential cell growth across the hypocotyl tip in dark-grown seedlings, which protects the underlying tissues and which opens during seedling development. We identified a small molecule that decelerates hook opening and found that it targets the protein RECEPTOR FOR ACTIVATED C KINASE 1A (RACK1A). We then showed that RACK1A promotes apical hook opening at the level of auxin signaling and response by adjusting differential cell growth. Our work paves the way to a better understanding of how plants regulate and adapt their growth during development.

In the natural environment, plants germinate and start developing in the dark surrounded by soil, from which they absorb moisture and nutrients. Dicotyledonous plants have evolved the apical hook, a transient structure at the hypocotyl apex that preserves the integrity of the shoot apical meristem during the early stages of development (1). The timing of the apical hook developmental process has been well characterized in *Arabidopsis thaliana* and three phases, named formation, maintenance, and opening phases, can be distinguished (2). The formation phase begins when hypocotyl bending starts and proceeds until maximum curvature is reached. During this phase, hook curvature is generated by differential cell elongation on either side of the hypocotyl as it grows, with the cells in the outer side of the hook elongating more than those in the inner side. The hook remains at its maximum level of curvature at the growing hypocotyl apex during the maintenance phase. Finally, the hypocotyl apex straightens, and the hook opens during the opening phase. During this phase, the cell elongation rate in the inner side of the hook increases until cell length is similar on both sides of the hypocotyl apex (2, 3).

Apical hook development is tightly regulated by multiple phytohormones, of which auxin and ethylene have been widely studied (4–6). An asymmetrical auxin response has been observed across the inner and outer sides of the hook, with an auxin response maximum present in the inner side (7). This auxin response gradient has been proposed to determine the differential cell elongation in the two sides of the hypocotyl apex that lead to hook formation and maintenance, with the high auxin response in the inner hook side repressing cell growth (6, 8). Similarly, loss of this auxin response maximum at the late maintenance phase releases cell growth repression in the inner hook side, leading to hook opening. Disruption of auxin synthesis, transport, or signaling through genetic mutations or pharmacological approaches consequently leads to impaired apical hook development (9–17).

The ubiquitin–proteasome system has been reported to play a prominent regulatory role in phytohormone signaling (18, 19). The ubiquitin-like protein RELATED TO UBIQUITIN/NEURAL PRECURSOR CELL EXPRESSED DEVELOPMENTALLY DOWN-REGULATED PROTEIN 8 (RUB/NEDD8) uses similar enzymatic machineries as ubiquitin for NEDD8 conjugation (neddylation) (20). AUXIN RESISTANT 1 (AXR1) is a subunit of a heterodimeric NEDD8-activating enzyme (21), and mutation in the *AXR1* gene impairs sensitivity to multiple phytohormones, including auxin (9, 14, 22, 23). Here, we applied a chemical biology screen to identify bioactive small molecules that target plant development, based on a difference in sensitivity between a mutant in *AXR1* and the wild type (WT), such as we have done previously (17), resulting in isolation of the compound DAPIA (Delay of Apical Hook Opening in *axr1-30*). By combining molecular genetics and biochemical approaches, we pinpoint a WD40 repeat scaffold protein, RECEPTOR FOR ACTIVATED C KINASE 1A (RACK1A), as the direct target of DAPIA and provide evidence that RACK1A modulates the auxin response gradient regulating differential cell growth during apical hook opening.

## Results

### Identification of the Small Molecule DAPIA, Which Affects Apical Hook Development in *Arabidopsis thaliana*.

**Fig. 1.**
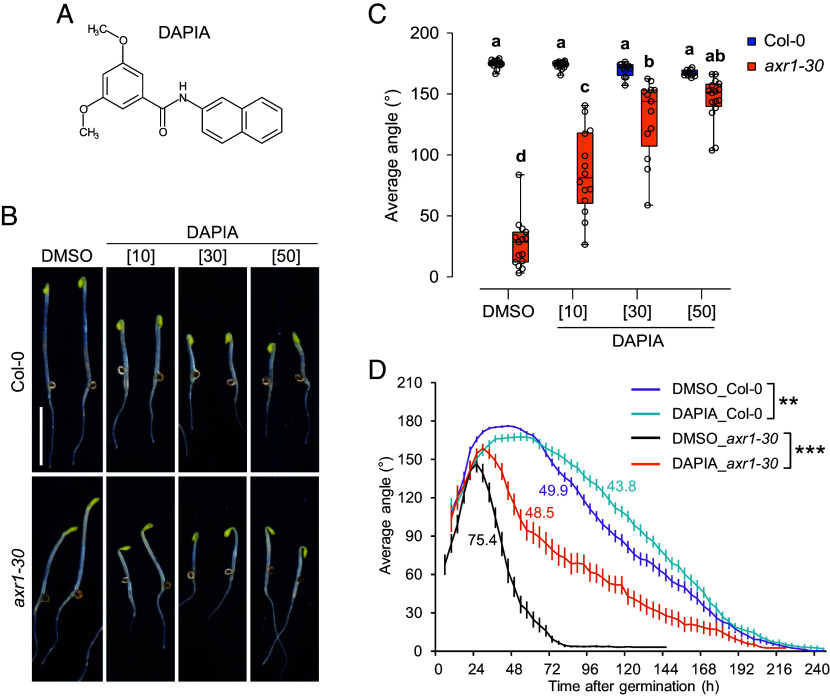
The compound DAPIA decelerates apical hook opening in *Arabidopsis* etiolated seedlings. (*A*) Chemical structure of DAPIA. (*B* and *C*) DAPIA dose–response analysis – representative images of apical hook phenotypes (*B*) and quantification of average hook angle (*C*) after 4 d of growth (including germination time) of Col-0 and *axr1-30* in darkness on medium supplemented with DMSO (mock) or DAPIA. Values in square brackets represent concentrations in µM. (Scale bar, 5 mm.) Data are shown as box plots and different letters indicate significantly different means of *N* = 8 to 17 seedlings at *P* < 0.05 (one-way ANOVA, Tukey’s multiple comparisons test) (*C*). (*D*) Kinematics of apical hook angle in Col-0 and *axr1-30* as measured every 4 h for 10 d of growth starting from germination (0 h) in darkness on medium supplemented with DMSO (mock) or 10 µM DAPIA. Error bars represent SEM; *N* = 23 to 61 seedlings. Color-coded values beside the curves represent the rate of early hook opening, expressed as the mean slope angle in degrees of the late maintenance-opening phase (from the maximum mean hook angle to the first mean hook angle below 30% of the maximum), for which asterisks indicate significant differences (Wilcoxon rank-sum test; **P < 0.01; ***P < 0.001).

We previously developed a screening strategy for the identification of compounds targeting differential plant growth and development, based on the difference in sensitivity between *Arabidopsis thaliana* Columbia-0 (Col-0) WT and the multiple phytohormone-insensitive mutant *axr1-30* (17). We used a similar strategy in the present work. From a screen of 4,560 diverse compounds, we obtained a small molecule that we named DAPIA (Fig. 1*A*), which was active in rescuing apical hook development in *axr1-30* in a dose-dependent manner, without obviously affecting the apical hook angle of WT, after 4 d of growth (including germination time) in darkness (Fig. 1 *B* and *C*). Reduced hypocotyl length was also noted in the etiolated seedlings grown in the presence of the compound, for both WT and *axr1-30* (Fig. 1*B*).

To further clarify the mode of action of DAPIA on apical hook development, we performed a kinematic analysis by continuously recording the apical hook angle of etiolated Col-0 and *axr1-30* seedlings, as previously described (14). We observed that DAPIA did have an effect in the WT, slightly delaying the formation phase as well as reducing the maximum hook angle and somewhat decelerating hook opening (Fig. 1*D*). As the mechanisms regulating the formation and opening phases of apical hook development are distinct (24), we chose to focus on the late maintenance-opening phase, during which differential growth is repressed, and equal growth is restored, across the apical hook sides. We calculated the hook opening rates during this phase (from the maximum mean hook angle to the first mean hook angle below 30% of the maximum) as the slope angles of the kinematic curves and performed statistical comparisons, revealing a highly significant deceleration of the early hook opening rate in the DAPIA-treated compared to the mock-treated WT (Fig. 1*D*). We then performed statistical comparison of the mock- and DAPIA-treated kinematic curves at the time points representing the late maintenance-opening phase in the mock control, which also revealed a significant effect of the compound during this phase in the WT (*SI Appendix*, Fig. S1*A*). The *axr1-30* mutant displayed a smaller maximum hook angle than the WT, no maintenance phase, and much more rapid hook opening than the WT in control conditions (Fig. 1*D*). DAPIA increased the maximum hook angle in *axr1-30* as well as strongly decelerating hook opening (Fig. 1*D*) and this effect of DAPIA in the opening phase was highly significant, both when comparing hook opening rates (Fig. 1*D*) and the kinematic curves (*SI Appendix*, Fig. S1*B*). Since germination takes approximately 21 to 36 h in our hands, the DAPIA dose–response data presented earlier at 4 d of growth (Fig. 1 *B* and *C*) corresponds to about 60 to 75 h after germination, at which time the WT is barely affected, while *axr1-30* is strongly affected, by DAPIA treatment (Fig. 1*D*). Together, these results suggest that DAPIA targets a mechanism promoting apical hook opening in the WT and that *axr1-30* may be more sensitive to this effect. As AXR1 lies upstream of multiple phytohormone signaling pathways, we speculate that defects in other non-DAPIA-targeted pathways regulating hook opening in *axr1-30* may lead to stronger DAPIA-induced repression of hook opening than in the WT.

To gain insight into DAPIA activity, we performed a structure–activity relationship (SAR) study. The chemical structure of DAPIA suggests possible cleavage, which would release an amide (DAPIA-N) or a carboxylic acid (DAPIA-C) (*SI Appendix*, Fig. S1*C*). These two potential metabolites, as well as several DAPIA analogs that we synthesized, were assayed for their capacity to affect apical hook development (*SI Appendix*, Fig. S1 *C* and *E*). DAPIA-N and DAPIA-C had no effect on apical hook angles of Col-0 or *axr1-30* after 4 d of growth (including germination time) in darkness (*SI Appendix*, Fig. S1*D*), suggesting the physiological effects of DAPIA are caused by the intact molecule rather than its metabolites. Similarly, none of the analogs tested exhibited an effect like that of DAPIA in rescuing *axr1-30* hook angle after 4 d, with most analogs having no effect at all, and only one analog, DAPIA-06, having a slight effect (*SI Appendix*, Fig. S1*E*). This suggests that DAPIA activity is linked to the whole molecule structure, as removals or modifications at various positions of the molecule generally abolish its bioactivity. The chemical stability of DAPIA was then tested by analyzing the presence of DAPIA-N and DAPIA-C in the growth medium and *in planta*. In growth medium containing DAPIA, neither of the two potential metabolites was detectable with or without the growth of seedlings on the medium (*SI Appendix*, Fig. S2*A*). Without plants, the concentration of DAPIA in the medium remained unchanged after treatment for 4 d. However, the presence of growing seedlings resulted in around a 50% reduction in the concentration of DAPIA in the medium, suggesting that DAPIA is efficiently taken up by the plants (*SI Appendix*, Fig. S2*A*). Only DAPIA and negligible concentrations of DAPIA-N were detectable in seedlings grown in the presence of DAPIA for 4 d (*SI Appendix*, Fig. S2 *A* and *C*). Overall, these results imply that DAPIA is chemically stable both in vitro and in planta.

### DAPIA Decelerates Apical Hook Opening and Requires the Auxin Signaling Components AXR2, ARF7, and ARF19 for This Effect.

**Fig. 2.**
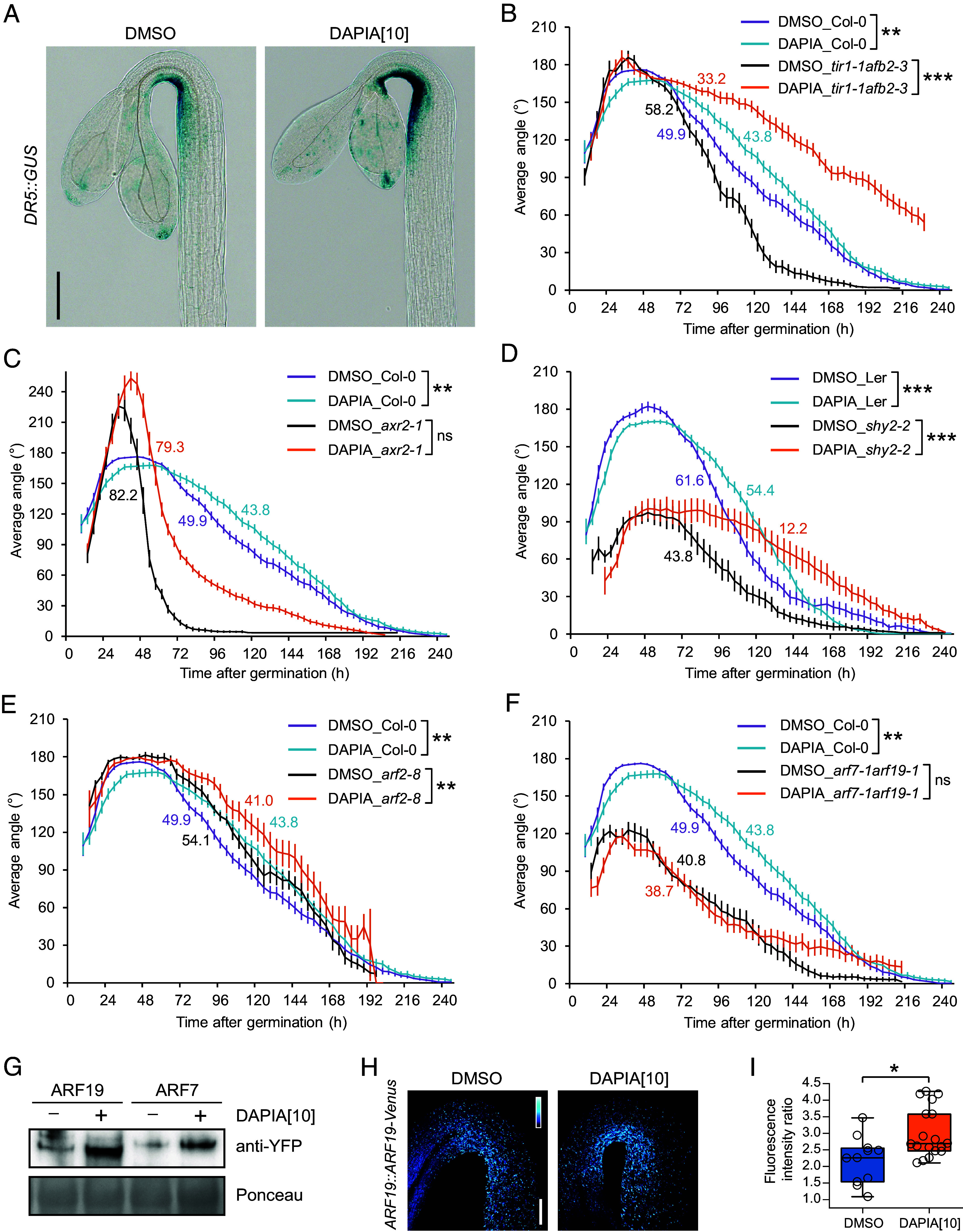
DAPIA affects the auxin response in the apical hook and requires the auxin signaling components AXR2, ARF7, and ARF19 to decelerate hook opening. (*A*) Representative images of apical hooks of GUS-stained *DR5::GUS* seedlings after 4 d of growth (including germination time) in darkness on medium supplemented with DMSO (mock) or DAPIA. Values in square brackets represent concentrations in µM. (Scale bar, 150 µm.) (*B*–*F*) Kinematics of apical hook angle in *tir1-1afb2-3* (*B*), *axr2-1* (*C*), *shy2-2* (*D*), *arf2-8* (*E*), and *arf7-1arf19-1* (*F*), together with the relevant WT, as measured every 4 h for 10 d of growth starting from germination (0 h) in darkness on medium supplemented with DMSO (mock) or 10 µM DAPIA. Error bars represent SEM; *N* = 16 to 61 seedlings. Color-coded values beside the curves represent the rate of early hook opening, expressed as the mean slope angle in degrees of the late maintenance-opening phase (from the maximum mean hook angle to the first mean hook angle below 30% of the maximum), for which asterisks indicate significant differences (Wilcoxon rank-sum test; ns–not significantly different; **P < 0.01; ***P < 0.001). (*G*) Immunoblot analysis of tissue samples from *ARF19::ARF19-Venus* and *ARF7::ARF7-Venus* seedlings, grown for 4 d in darkness on medium supplemented with DMSO (mock) or 10 µM DAPIA, probed with anti-YFP antibody. Ponceau stain was used as loading control. (*H* and *I*) Representative confocal images (*H*) and quantification of Venus fluorescence gradient (inner:outer side average fluorescence intensity ratio) (*I*) of apical hooks of dark-grown *ARF19::ARF19-Venus* seedlings at the late maintenance-opening phase. Seedlings were grown on medium supplemented with DMSO (mock) or 10 µM DAPIA. (Scale bar, 100 µm.) Low-to-high Venus signal intensity is represented as a blue-to-white color gradient according to the inset, and maximum intensity projections of z-stacks are shown (*H*). Data are shown as box plots and asterisks indicate significantly different means of *N* = 11 to 18 seedlings (Wilcoxon rank-sum test; *P < 0.05) (*I*). Values in square brackets represent concentrations in µM.

The asymmetric auxin response that is required for apical hook development includes an auxin response maximum in the inner side of the hook (14). In contrast to the effects of DAPIA on apical hook development (Fig. 1*D*), exogenous auxin treatment abolishes hook formation in dark-grown seedlings (15), suggesting that DAPIA is physiologically distinct from auxin. However, we found that the application of DAPIA resulted in an enlarged region of auxin response maximum in the inner side of the hook of the WT after 4 d of growth (including germination time) in darkness, as observed via *DR5::GUS* auxin response reporter expression (Fig. 2*A*). Thus, DAPIA may exert its effect on decelerating hook opening via locally enhancing the auxin response maximum in the inner side of the hook. Alternatively, since 4 d of growth corresponds to about 60 to 75 h after germination as described earlier, DAPIA may rather maintain the auxin response maximum in the inner side, which should be starting to reduce in the untreated control at this late maintenance-opening phase time point (Fig. 1*D*).

To investigate possible involvement of auxin signaling in DAPIA activity, we performed hook angle kinematic analysis in various auxin perception- and signaling-defective mutants in the absence or presence of DAPIA. Compared to the Col-0 WT, the auxin receptor mutant *tir1-1afb2-3* exhibited a slightly exaggerated maximum hook angle, followed by earlier, more rapid hook opening (Fig. 2*B*). The application of DAPIA strongly decelerated hook opening in *tir1-1afb2-3*, resulting in a highly significant effect of the compound on the late maintenance-opening phase hook opening rate (Fig. 2*B*) and on the kinematic curve itself during this phase (*SI Appendix*, Fig. S3*A*). The reasons for this oversensitivity of *tir1-1afb2-3* to the effect of DAPIA on hook opening compared to the WT are not clear, but as for *axr1-30*, might possibly be explained by an upstream role of the TIR1/AFB receptors in multiple auxin signaling pathways involved in hook opening.

The AUX/IAA gain-of-function mutant *axr2-1/iaa7* exhibited an exaggerated hook, no maintenance phase, and much faster hook opening than the Col-0 WT in control conditions (Fig. 2*C*). The main effect of DAPIA in *axr2-1* was to enhance the already exaggerated hook, leading to a significant effect on the kinematic curve during the opening phase (*SI Appendix*, Fig. S3*B*). However, the rate of hook opening during this phase was not significantly affected by DAPIA treatment in *axr2-1* (Fig. 2*C*), revealing this mutant to be resistant to the deceleration effect of DAPIA on the opening process. Much like for Col-0, hook formation was slightly delayed, maximum hook angle was decreased and hook opening was somewhat decelerated by DAPIA treatment in the Landsberg *erecta* (Ler) WT, leading to a highly significant effect on the late maintenance-opening phase hook opening rate (Fig. 2*D*) as well as a significant effect on the kinematic curve (*SI Appendix*, Fig. S3*C*). The AUX/IAA gain-of-function mutant *shy2-2/iaa3* exhibited a much smaller maximum hook angle than the Ler WT as well as slower hook opening in control conditions (Fig. 2*D*). DAPIA treatment extended the maintenance phase of *shy2-2* as well as significantly decelerating the hook opening rate (Fig. 2*D*), leading to a significant effect on the kinematic curve (*SI Appendix*, Fig. S3*D*), during the late maintenance-opening phase.

We next tested the activity of DAPIA on *arf2-8*, a loss of function mutant in the transcriptional modulator ARF2 shown to be involved in apical hook development through the ethylene signaling pathway (7), which displayed similar hook angle kinematics to the Col-0 WT in control conditions (Fig. 2*E*), as shown previously (25). Although DAPIA treatment did not significantly affect the kinematic curve during the late maintenance-opening phase in *arf2-8* (*SI Appendix*, Fig. S3*E*), the compound did significantly reduce the hook opening rate during this phase (Fig. 2*E*). Finally, *arf7-1arf19-1*, a loss-of-function mutant in the auxin transcriptional activators ARF7 and ARF19, showed a strong impairment in hook formation, as shown previously (15), as well as slower hook opening compared to the Col-0 WT in control conditions (Fig. 2*F*). Moreover, *arf7-1arf19-1* was strongly resistant to the effects of DAPIA on the late maintenance-opening phase hook opening rate (Fig. 2*F*) and kinematic curve (*SI Appendix*, Fig. S3*F*). Taken together, these results suggest that DAPIA requires the functional auxin signaling components AXR2, ARF7, and ARF19 to exert its effect on decelerating apical hook opening.

Since *arf7-1arf19-1* displays somewhat slower hook opening than the WT and shows a striking resistance to DAPIA, indicating requirement of ARF7 and ARF19 for DAPIA to exert its effect on decelerating apical hook opening, these transcription factors seem very likely to play a role in the opening process. We were curious as to what effect DAPIA might have on the abundance of ARF7 and ARF19 at the protein level. We therefore analyzed tissue samples from *ARF7::ARF7-Venus* and *ARF19::ARF19-Venus* etiolated seedlings grown in the presence of DAPIA for abundance of the Venus protein by Western blotting, revealing a strong increase in protein abundance compared to the mock control at the whole seedling level (Fig. 2*G*). To investigate this effect specifically in the apical hook, we performed confocal microscopy imaging of etiolated *ARF19::ARF19-Venus* seedlings grown in the presence of DAPIA, revealing a fluorescence signal maximum in the inner hook side and a clear DAPIA-induced enhancement of this maximum at the late maintenance-opening phase (Fig. 2*H*). Quantification of the fluorescence signal across the hook revealed that DAPIA enhanced an inner:outer side fluorescence intensity ratio, implying the enhancement of an ARF19 protein abundance gradient, across the hook (Fig. 2*I*). This is likely to be a cause of the DAPIA-enhanced/maintained auxin response gradient across the hook, which consequently disrupts hook opening.

### RACK1A Is a Direct Target of DAPIA.

**Fig. 3.**
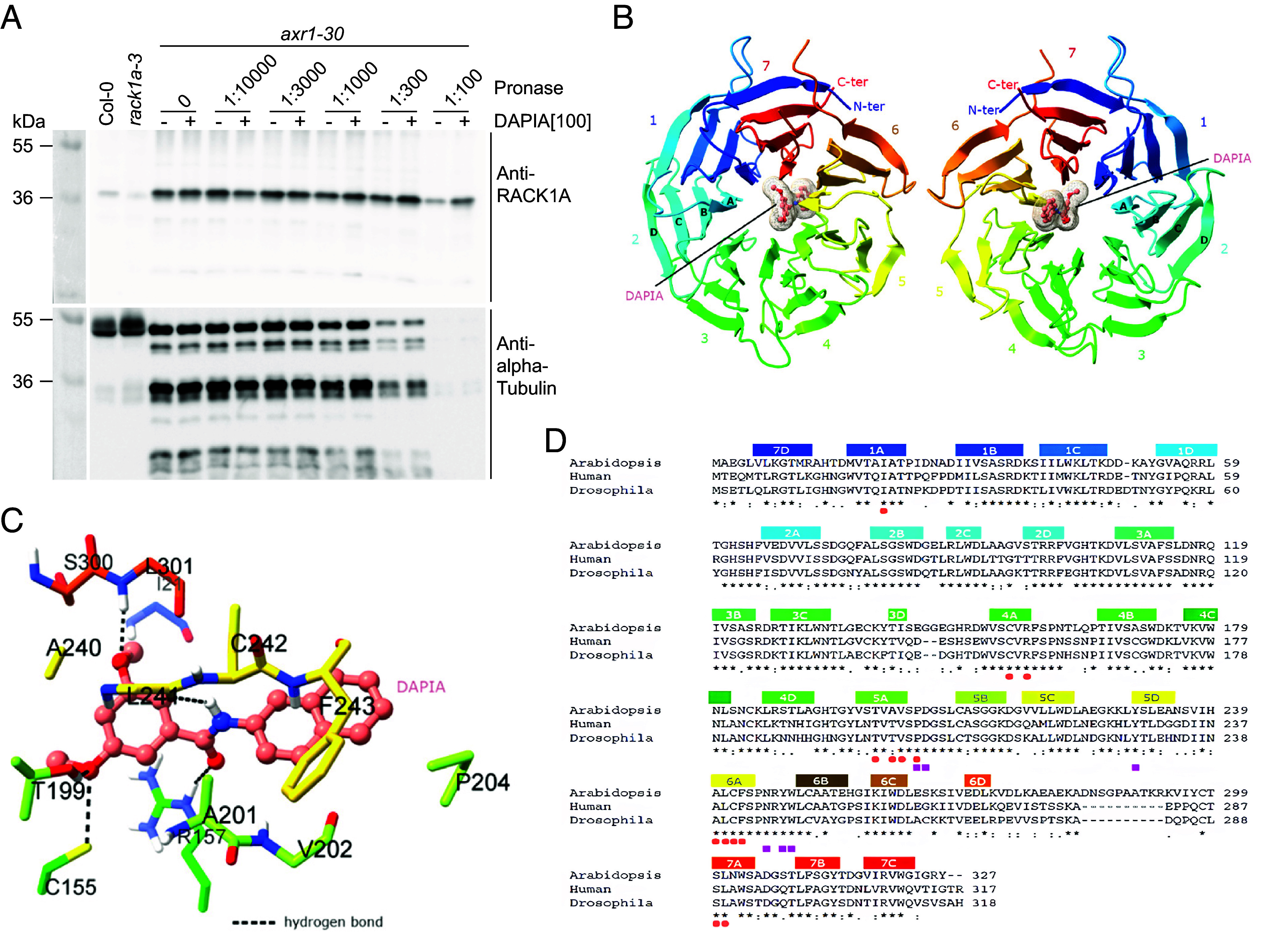
RACK1A is the direct target of DAPIA. (*A*–*C*) Direct binding between DAPIA and RACK1A validated by proteolytic analysis (*A*) and molecular docking (*B* and *C*). (*A*) Total protein extracts from 4-d-old etiolated seedlings of *axr1-30*, as well as Col-0 and *rack1a-3* as controls, were incubated with DMSO (mock) or DAPIA and subjected to various dilutions of pronase (expressed as total enzyme:total protein ratios). RACK1A protein levels were detected through protein gel blot analysis using anti-RACK1A antibody (*Upper* blot) and anti-α-Tubulin antibody (*Lower* blot) as a reference protein control. Each blot has its own reference ladder, viewed with a different mode and therefore saved as a separate image. Values in square brackets represent concentrations in µM. (*B*) *Top* (*Left* panel) and bottom (*Right* panel) views of RACK1A 3D structure with the DAPIA binding site predicted by the docking simulation. RACK1A, in the ribbon diagram, adopts a β-propeller conformation. The seven blades of the β-propeller are numbered 1-7 from the N terminus (N-ter, blue) to the C terminus (C-ter, red) in rainbow mode. The four antiparallel β-strands in each blade are labeled A–D, as exemplified in blade 2. The small molecule DAPIA is bound in a cavity in the central channel and is shown in stick-and-ball and surface mesh modes in salmon color. (*C*) A close-up view of DAPIA in the predicted binding pocket of RACK1A. The thirteen RACK1A residues involved in the interaction with DAPIA are shown in sticks, with the same color codes as used in *B*. Hydrogen bonds are shown as black dashed lines. (*D*) Multiple sequence alignment of *Arabidopsis*, human, and *Drosophila* RACK1 in relationship with the 3D structure of *Arabidopsis* RACK1A docked with DAPIA. Protein sequences were obtained from UniProt using the following accession numbers: *Arabidopsis thaliana* RACK1A: O24456, *Homo sapiens* (human) RACK1: P63244, *Drosophila melanogaster* RACK1: O18640. Sequences are aligned using Clustal Omega. Secondary structural elements of *Arabidopsis* RACK1A (four β-strands in each of the seven blades) are indicated above the sequences, with the same alphanumeric and color codes as used in *B*. Invariant amino acids are denoted by asterisks. The DAPIA-interacting residues are indicated by salmon circles and those constituting the conserved region 2 by purple squares.

To identify the protein target of DAPIA, we performed a de novo drug affinity responsive target stability (DARTS) assay (26), a technology based on the evidence that some proteins, when bound to chemical ligands, are protected from degradation by proteases. We reasoned that the stronger effect of DAPIA on decelerating hook opening in *axr1-30* compared to the WT (Fig. 1*D*) might enable easier identification of the protein target of DAPIA in the mutant. We thus incubated total protein extracts from *axr1-30* etiolated seedlings with DAPIA and treated with the proteolytic enzyme pronase before subsequent analysis by LC–MS/MS, leading to the identification of several protein candidates as being protected from digestion and therefore potential targets of DAPIA (Dataset S1). We chose to focus on one particular candidate, RACK1A, as it is known to be involved in multiple phytohormone signaling pathways (27), but has not previously been implicated in the regulation apical hook development. This protein is the *Arabidopsis* homologue of the tobacco WD40 repeat ArcA, described as a scaffold protein involved in glucose, gibberellin, and abscisic acid signaling pathways (27, 28). To validate the binding ability of DAPIA with RACK1A, we tested the effects of DAPIA on pronase proteolytic degradation of RACK1A protein by immunoblotting in the *axr1-30* protein extracts. DAPIA started to show protection of RACK1A against proteolysis when the pronase:protein ratios were in the range of 1:1000 to 1:100, with the highest pronase level displaying the most obvious difference between DAPIA-treated and nontreated samples (Fig. 3*A*). These results provide support for an interaction between DAPIA and RACK1A in vitro.

Next, we performed molecular docking analysis to explore the likely interaction between DAPIA and RACK1A and to predict the binding site. We docked DAPIA in silico to the available structure of *Arabidopsis* RACK1A (Protein Data Bank (PDB) code 3DM0) (29). The overall protein structure adopts a seven-bladed β-propeller fold circularly arranged around a central channel with a diameter of ~9Å, which is canonical for the WD40 family proteins (29). Each propeller blade is composed of four antiparallel β-strands, named A to D. Dockings with DAPIA predicted only one specific binding site within the central channel of the β-propeller fold with a reasonable estimated free energy of binding of −9.28 kcal mol^−1^ (Fig. 3*B*). According to the interaction mode, DAPIA formed hydrogen bond interactions with Cys155, Arg157, Thr199, Leu241, and Leu301, and established extensive beneficial contacts with Ile21, Cys155, Arg157, Thr199, Ala201, Val202, Pro204, Ala240, Leu241, Cys242, Phe243, Ser300, and Leu301 (Fig. 3 *B* and *C*). This simulation suggests that DAPIA may tightly bind to RACK1A via the binding pocket located in the protein’s central channel. The thirteen amino acid residues constituting the binding pocket are also conserved among *Arabidopsis*, human, and *Drosophila*, with the exception of Ala201, which is replaced by Thr in the latter two species (Fig. 3*D*) (29). Further analysis of the spatial distribution of these 13 amino acids revealed that they are all located at the inner β-strand A, involving blades 1 and 4 to 7, except for Pro204 that is positioned in the loop connecting strand A and B of blade 5. Moreover, eight of them are from blades 5 and 6 (four each). Previous studies revealed two conserved surface regions within the RACK1 proteins; conserved region 1 is located on the top face of the propeller and region 2 on the bottom. They impart a scaffolding function to the protein by mediating protein–protein interactions (29). Conserved region 2 is mainly composed of amino acids from blade 5 (Pro204, Asp205, and Tyr230) and blade 6 (Asn246, Tyr248, and Trp249) (29). This result implies that β-propeller blades 5 and 6 may play an important role in facilitating interactions with the ligand and with other partner proteins. To summarize, our docking simulations suggest that DAPIA may target RACK1A through extensive interactions with a binding site buried in the central channel, which consequently may interfere with protein–protein interactions mediated by the conserved region 2 of RACK1A.

To confirm the docking result qualitatively and quantitatively, we characterized the specificity and quantified the kinematics and affinity of binding between DAPIA and RACK1A using microscale thermophoresis (MST). The inactive analogs DAPIA-02 and DAPIA-07 were used as negative controls. In the absence of Tween 20 or protease inhibitor cocktail (PIC), the signal to noise (S/N) value of DAPIA-treated samples reached 46.9, implying good binding between DAPIA and RACK1A (*SI Appendix*, Fig. S4*A*), while the presence of Tween 20 or PIC abolished the binding (S/N value 1.4) (*SI Appendix*, Fig. S4*B*). The dose–response curves further revealed a dissociation constant (*K*_d_) of 32.8 ± 15.7 μM (SD), suggesting again the optimal binding affinity of DAPIA to RACK1A (*SI Appendix*, Fig. S4*C*). Notably, the two inactive analogs, DAPIA-02 and DAPIA-07, did not show any binding affinity with RACK1A (*SI Appendix*, Fig. S4*D*). These results confirm that RACK1A is a direct target of DAPIA.

### RACK1A Positively Regulates Apical Hook Opening by Suppressing the Auxin Response Gradient.

**Fig. 4.**
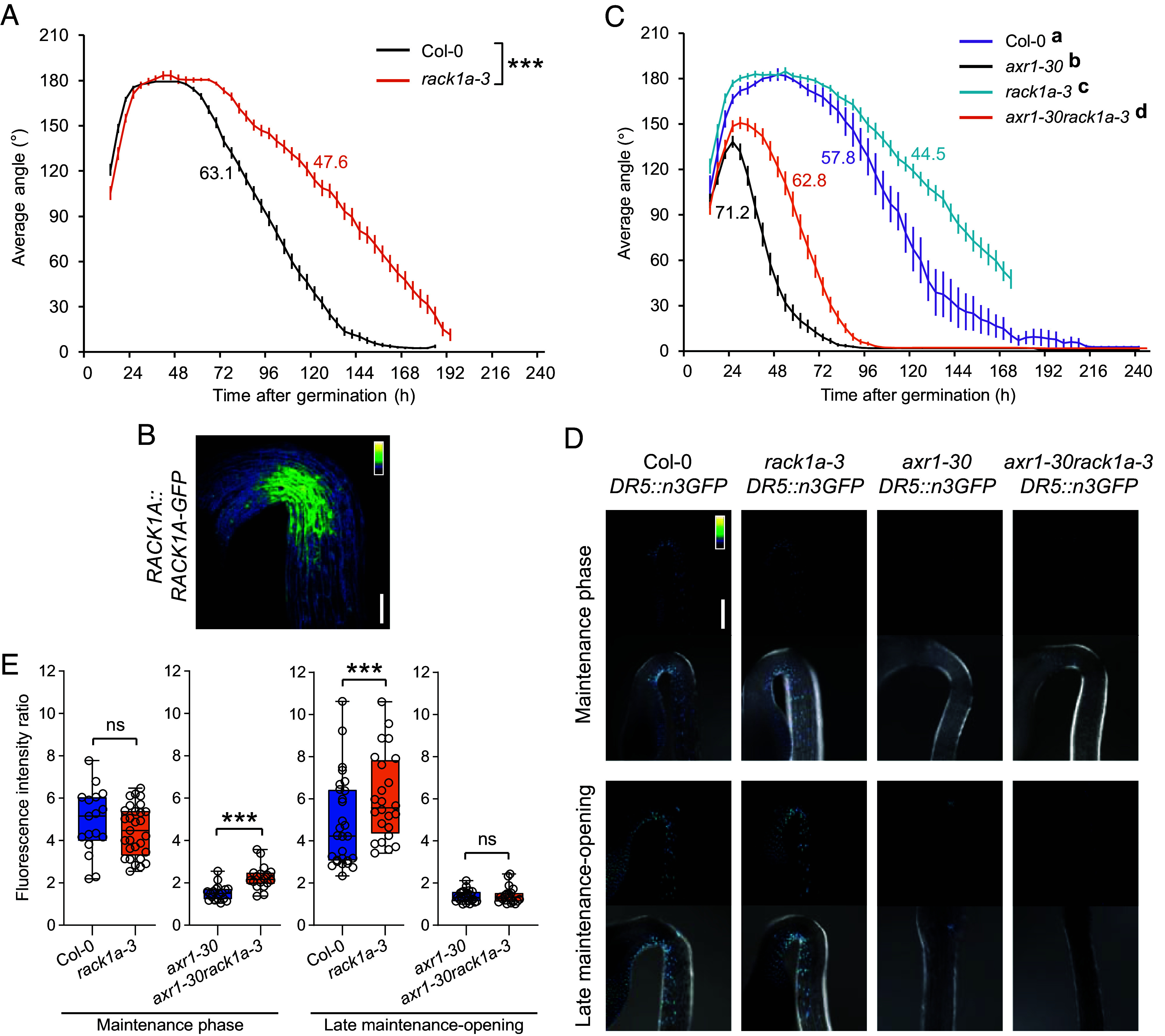
RACK1A regulates apical hook opening at the level of auxin response. (*A*) Kinematics of apical hook angle in Col-0 and *rack1a-3* as measured every 4 h for 10 d of growth starting from germination (0 h) in darkness. (*B*) Representative confocal image of the apical hook of dark-grown *RACK1A::RACK1A-GFP* seedling at the late maintenance-opening phase. (*C*) Kinematics of apical hook angle in Col-0, *axr1-30*, *rack1a-3*, and *axr1-30rack1a-3* as measured every 4 h for 10 d of growth starting from germination (0 h) in darkness. Error bars represent SEM; *N* = 11 to 46 seedlings (*A* and *C*). Color-coded values beside the curves represent the rate of early hook opening, expressed as the mean slope angle in degrees of the late maintenance-opening phase (from the maximum mean hook angle to the first mean hook angle below 30% of the maximum), for which asterisks (*A*) or different letters (*C*) indicate significant differences (Wilcoxon rank-sum test; ***P < 0.001; different letters: *P* < 0.05). (*D* and *E*) Representative confocal images (*D*) and quantification of auxin response gradient (inner:outer side average nuclear GFP fluorescence intensity ratio) (*E*) of apical hooks of dark-grown *DR5::n3GFP* seedlings in Col-0, *axr1-30*, *rack1a-3,* and *axr1-30rack1a-3* backgrounds at time points representing the maintenance phase and late maintenance-opening phase of the WT. [Scale bar, 100 µm (*B*) and 200 µm (*D*).] Low-to-high GFP signal intensity is represented as a blue-to-yellow color gradient according to the insets and maximum intensity projections of z-stacks are shown (*B* and *D*). Data are shown as box plots and asterisks indicate significantly different means of *N* = 17 to 29 seedlings (Wilcoxon rank-sum test; ns–not significantly different; ***P < 0.001) (*E*).

Our next step was to perform hook angle kinematic analysis in the loss-of-function mutant *rack1a-3*. In control conditions, *rack1a-3* displayed a similar maximum angle of hook curvature to the WT, but a longer maintenance phase and a much slower rate of early hook opening (Fig. 4*A*). The difference between the *rack1a-3* and Col-0 hook opening rates was highly significant at the late maintenance-opening phase (Fig. 4*A*), as was the difference between their kinematic curves at this phase (*SI Appendix*, Fig. S5*A*). In fact, the effect of the *rack1a-3* mutation on the late maintenance-opening phase was stronger than that of DAPIA treatment in the WT (*SI Appendix*, Fig. S5*B*). These results support the idea that DAPIA decelerates hook opening by targeting RACK1A, suggesting that RACK1A is involved in regulating hook opening. Unlike the effect of DAPIA treatment in the WT control (*SI Appendix*, Fig. S5 *B* and *C*), DAPIA treatment of *rack1a-3* resulted in a much smaller maximum angle of hook curvature and a slightly shorter maintenance phase than in mock conditions (*SI Appendix*, Fig. S5*B*), leading to a highly significant effect on the late maintenance-opening phase kinematic curve (*SI Appendix*, Fig. S5*D*). However, importantly, the rate of hook opening in *rack1a-3* was not significantly affected by DAPIA treatment in this phase (*SI Appendix*, Fig. S5*B*). The resistance of the hook opening rate of *rack1a-3* to deceleration by DAPIA strongly suggests that DAPIA affects hook opening by targeting RACK1A. Nevertheless, the inhibition of hook formation in *rack1a-3* by DAPIA suggests that DAPIA may target alternative proteins involved in hook formation, rather than opening, in the absence of RACK1A. However, it is likely that RACK1A is the preferred target of DAPIA when this protein is present since this could explain why the effects of DAPIA on the formation phase of the WT were rather mild and somewhat inconsistent (Fig. 1*D* and *SI Appendix*, Figs. S3*C* and S5*C*).

In *Arabidopsis*, there are two other members of the *RACK1* gene family, *RACK1B* and *RACK1C*, which display high sequence similarity with *RACK1A* (30). We reasoned that RACK1B and RACK1C may be functionally redundant with RACK1A in the regulation of hook opening and therefore attempted to generate double mutants of *rack1a-3* with *rack1b-2* and *rack1c-1* by genetic crossing. Although we could not identify seedlings at the following generations that were simultaneously homozygous for both *rack1a-3* and *rack1b-2*, possibly due to early embryo lethality in this particular allele combination, or unknown genetic factors, we experienced no such problems in generating double homozygous *rack1a-3rack1c-1*. We proceeded to analyze hook angle kinematics in this double mutant as well as *rack1b-2rack1c-1* and the three single mutants (*SI Appendix*, Fig. S6*A*). Both *rack1a-3* and *rack1b-2* single mutants displayed decelerated hook opening, leading to significant differences to the WT in both hook opening rates and kinematic curves at the late maintenance-opening phase (*SI Appendix*, Fig. S6 *A* and *B*). While *rack1c-1* displayed only a slight deceleration of hook opening compared to the WT and was not significantly different to the WT at the late maintenance-opening phase in either hook opening rate or kinematic curve (*SI Appendix*, Fig. S6 *A* and *B*), neither was this mutant significantly different to the other single mutants at this phase (*SI Appendix*, Fig. S6 *A* and *C*), suggesting that *rack1c-1* is somewhat affected in hook opening. Interestingly, the combination of *rack1a-3* and *rack1c-1* resulted in an additive effect, in which the double mutant was strongly reduced in the hook opening rate and highly significantly different to the *rack1a-3* and *rack1c-1* single mutants in both hook opening rates and kinematic curves at the late maintenance-opening phase (*SI Appendix*, Fig. S6 *A*, *D*, and *E*). These results suggest that RACK1C plays a functionally redundant role with RACK1A in the promotion of apical hook opening. Although it is difficult to make conclusions about a potentially redundant role of RACK1B with RACK1A in hook opening without the *rack1a-3rack1b-2* double mutant, the hook opening rate was unaffected in *rack1b-2rack1c-1* compared to the *rack1b-2* and *rack1c-1* single mutants (*SI Appendix*, Fig. S6 *A*, *F*, and *G*), implying that RACK1A is likely the predominant regulator of apical hook opening among the three RACK1 proteins.

To further investigate these findings, we next analyzed hook angle kinematics in the estradiol-inducible triple RACK1A, B, and C-targeted artificial microRNA mutant *amiR-rack1-es1*. Under estradiol treatment, *amiR-rack1-es1* displayed a delay in hook formation and opening compared to the WT (*SI Appendix*, Fig. S7*A*), which resulted in a highly significant difference to the WT in both hook opening rate and kinematic curve at the late maintenance-opening phase (*SI Appendix*, Fig. S7 *A* and *B*). However, surprisingly, the triple mutant was not more affected in this phase than *rack1a-3* (*SI Appendix*, Fig. S7 *A* and *C*). We next investigated the effects of DAPIA on hook angle kinematics in the estradiol-induced triple mutant. The main effect was to decrease the maximum angle of hook curvature (*SI Appendix*, Fig. S7*D*), which led to a significant effect on the kinematic curve of the late maintenance-opening phase (*SI Appendix*, Fig. S7*E*), but no change in the rate of hook opening at this phase (*SI Appendix*, Fig. S7*D*), suggesting resistance of *amiR-rack1-es1* to deceleration of hook opening by DAPIA. Using quantitative real-time PCR, we then showed that although silencing of the three *RACK1* genes was induced by estradiol in our experimental conditions in the inducible triple mutant, some low-level expression of all three genes was still detectable (*SI Appendix*, Fig. S8*A*). This may possibly explain why the induced triple mutant was not more strongly affected in hook opening than *rack1a-3*. Taken together, our results support the proposal that DAPIA decelerates apical hook opening through targeting of the RACK1A protein, implying a positive role of RACK1A in hook opening, for which the other RACK1 proteins are likely to be functionally redundant. Similarly to *rack1a-3*, *amiR-rack1-es1* showed some sensitivity to the effects of DAPIA treatment on the phase of hook formation, upholding the idea that there may also be other molecular pathways targeted by DAPIA as mentioned earlier, which is supported by the identification of several other potential protein targets of this compound in our DARTS assay (Dataset S1).

Since our results suggest that RACK1A is involved in regulating apical hook opening, we next investigated the localization pattern of the RACK1A protein in apical hooks, by observing fluorescence signal at the late maintenance-opening phase in etiolated seedlings of the *RACK1A::RACK1A-GFP* reporter line. A cytosolic fluorescence signal was observed throughout the hook region, but the signal was particularly strong at the inner hook side (Fig. 4*B*). This result implies that *RACK1A* is highly expressed in the inner hook side at the onset of the opening process, supporting the idea that RACK1A is involved in suppressing the auxin gradient across the hook at the end of the maintenance phase and thereby positively regulating hook opening.

Considering that DAPIA partially rescues the early-opening apical hook phenotype of *axr1-30* (Fig. 1*D*), we hypothesized that mutations in RACK1A should achieve a similar result. To test this hypothesis, we genetically crossed *axr1-30* and *rack1a-3* and performed hook angle kinematic analysis in the double homozygous mutant, revealing a distribution that was intermediate between that of the single mutants at the maintenance and opening phases (Fig. 4*C*). Furthermore, statistical analysis confirmed that the *axr1-30* mutant’s hook opening rate and kinematic curve were statistically significantly rescued toward a WT-like hook angle kinematics phenotype at the late maintenance-opening phase by addition of the *rack1a-3* mutation (Fig. 4*C* and *SI Appendix*, Fig. S8*B*). Since our results suggest that DAPIA can enhance or maintain the auxin response in the apical hook at the end of the maintenance phase, resulting in deceleration of hook opening, we hypothesized that mutations in RACK1A should achieve the same result. As the early-opening apical hook phenotype of the *axr1-30* mutant is partially rescued by addition of the *rack1a-3* mutation (Fig. 4*C*), we also wondered what effect mutations in *RACK1A* would have on the auxin response in the hook of this mutant. To investigate this, we introduced the auxin response reporter line *DR5::n3GFP* into the *rack1a-3* and *axr1-30rack1a-3* backgrounds. We examined the auxin response gradient across the apical hook by observing the *DR5::n3GFP* signal at both the maintenance and late maintenance-opening phases of the WT (around 45 to 55 and 60 to 75 h after germination, at which time points hook opening was significantly affected in *axr1-30rack1a-3* compared to *axr1-30* (in Fig. 4*C*), and in *rack1a-3* compared to the WT (in Fig. 4 *A*), respectively). At the earlier time point, there was no difference in the inner:outer side auxin response gradient across the apical hook between the WT and *rack1a-3* (Fig. 4 *D* and *E*). The *axr1-30* mutant showed a much lower auxin response gradient across the hook than the WT, which agrees with its early hook opening phenotype (Fig. 4 *D* and *E*). This low gradient was partially rescued at the earlier time point by the introduction of *rack1a-3*, being slightly but significantly increased in *axr1-30rack1a-3* compared to *axr1-30* (Fig. 4 *D* and *E*). This result agrees with the partial rescue of the *axr1-30* early hook opening phenotype at this time point by combination with *rack1a-3* that was shown earlier (Fig. 4*C*). At the later time point, *rack1a-3* displayed a significantly increased auxin response gradient compared to the WT, while the auxin response gradients in *axr1-30* and *axr1-30rack1a-3* were no longer different (Fig. 4 *D* and *E*). These results support the idea that RACK1A positively regulates apical hook opening by suppressing the auxin response gradient in the hook.

Collectively, our results suggest that RACK1A plays a positive role in apical hook opening by negatively regulating the local auxin response maximum in the inner side of the hook via an AXR2/ARF7/ARF19 auxin signaling pathway. In this way, RACK1A acts at the transition between the hook maintenance and opening phases to suppress differential growth and reestablish equal growth across both sides of the hook, inducing its opening. To confirm the roles of ARF7 and ARF19 in this process, we attempted to cross *rack1a-3* with the *ARF7::ARF7-Venus* and *ARF19::ARF19-Venus* reporter lines. However, upon genotyping over 150 seedlings preselected for the presence of a Venus signal, none of them were found to be simultaneously homozygous for both *rack1a-3* and the relevant reporter line. The reasons for this are unclear but it may be that unknown genetic interactions prevent both the construct and mutation from being simultaneously homozygous. In any case, it will be interesting to further investigate the details of the involvement of these ARF transcription factors, the AUX/IAA AXR2, and other yet unknown mechanisms in RACK1A-mediated apical hook opening in future studies.

## Discussion

Phytohormones and their crosstalk mediate the morphological adaptability and plasticity of plants that are essential for their survival and development. Although some crucial modulators involved in phytohormone signaling have been identified through genetic approaches (7, 31–33), many components remain unknown due to gene redundancy or loss-of-function lethality and complex interconnected crosstalk. The isolation of small, bioactive molecules has proven to be an effective approach toward the unraveling of complex phytohormone signaling pathways while overcoming many of these issues (17, 34–42). Despite many such effective approaches, few of them have reported the successful identification of the direct targets of the compounds. In this work, we isolated the small molecule DAPIA and unveiled its direct molecular target RACK1A, a WD40 repeat scaffold protein, as a modulator of apical hook opening at the level of auxin signaling and response. Among the three genes encoding RACK1 proteins in *Arabidopsis*, *RACK1A* shows the highest expression in various tissues and organs (43), implying its predominant role in the regulation of various developmental processes. Our work suggests that RACK1A may regulate apical hook opening in a functionally redundant manner together with RACK1B and C, but that RACK1A is probably the predominant regulator of this process among the three proteins. Interestingly, RACK1A has also very recently been shown to positively regulate hypocotyl elongation in etiolated *Arabidopsis* seedlings (44), which agrees with the reduction in hypocotyl elongation we observed in dark-grown seedlings treated with DAPIA (Fig. 1*B*).

Much of the work that has unraveled essential roles for phytohormones in apical hook development has been focused on the formation phase, during which the establishment of a precise auxin gradient across the hook is essential. Although hook opening requires subsequent reduction of this gradient, the signaling pathways regulating the appearance and disappearance of the auxin maximum appear to be distinct. For example, experimental evidence suggests that intercellular auxin transport mechanisms are involved in the establishment of the auxin maximum required for hook formation, while the depletion of the auxin maximum required for hook opening mainly involves intracellular alterations of auxin (24). We therefore took advantage of the effect of DAPIA in decelerating apical hook opening to investigate the molecular events involving RACK1A that govern this poorly studied phase of hook development, revealing that RACK1A likely promotes hook opening via mechanisms involving AXR2, ARF7, and ARF19 signaling, at the level of auxin response. Our results imply that RACK1A negatively regulates the abundance of ARF7 and ARF19 as well as the auxin response maximum in the inner hook side, leading to reestablishment of equal growth across the hook, resulting in its opening.

RACK1A is a versatile scaffold protein that has already been shown to be involved in multiple phytohormonal signaling pathways, including that of auxin (27). Our work suggests that the signaling pathways in which RACK1A is involved, that positively regulate hook opening, do so by negatively regulating the auxin response maximum found in the inner side of the hook, which is likely a consequence of enhanced ARF7 and ARF19 abundance in this region. In contrast, previous studies have reported a positive association between RACK1A and auxin response in the control of lateral root development (30, 45). These opposing findings suggest that RACK1A may mediate auxin response in a flexible way, depending on the cell/tissue type and timing of development, implying a role in spatiotemporal regulation of the ARF machinery. Interestingly, although our work shows that RACK1A enhances ARF7 and ARF19 protein abundance in the hook, RACK1A has been proposed as a down-regulator of auxin signaling during salinity acclimation (46). The apparent flexibility of auxin signaling components, that may interact in different ways depending on the developmental context, suggests an effective, adaptable means by which plants ensure plasticity, with appropriate developmental responses to complex signaling networks. Considering that RACK1A is a scaffold protein, it likely integrates unknown interactors to modulate signaling during its regulation of the auxin response in the apical hook. Identification of such missing components will facilitate a better understanding of the mechanisms mediating differential growth during apical hook development.

## Materials and Methods

For detailed information on all procedures see *SI Appendix*, *Supporting Information Text*. All raw data, protocols, and materials may be accessed by contacting the corresponding author.

### Plant Materials, Growth Conditions, Chemicals, and Kinematic Analysis.

All *Arabidopsis thaliana* lines used were published previously, except for the double *rack1a-3rack1c-1* mutant and multiple genotype combinations of *axr1-30*, *rack1a-3,* and *DR5::n3GFP*, which were assembled by genetic crossing. Seeds were sown on solid growth medium supplemented with indicated concentrations of chemicals. Assays were performed with etiolated seedlings grown in darkness at 22 °C. The chemical screen was conducted as described previously (17) and DAPIA was isolated as ChemBridge molecule 5327372. Stock solutions of chemicals were prepared in DMSO. For kinematic analysis, seedlings were grown at 22 °C in a dark box illuminated with infrared light from 850 nm LEDs, photographed every hour for 10 d, and quantified for hook angle using ImageJ (Fiji) software every 3 or 4 h starting 12 h after germination.

### GUS Staining, Confocal Microscopy, and Venus Immunoblotting.

GUS staining was performed as previously described (47). Fluorescence imaging was performed with Zeiss LSM 800 and 880 confocal microscopes. Rabbit anti-YFP antibody (Agrisera) at 1:5000 was used for ARF7-Venus and ARF19-Venus detection by immunoblotting.

### DARTS Assay and Tryptic Peptide Analysis.

The DARTS assay was performed as described previously (48). Trypsin-digested samples were analyzed by LC–MS/MS and the resulting spectra were searched against the *Arabidopsis* TAIR10 database.

## Supplementary Material

Appendix 01 (PDF)

Dataset S01 (XLSX)

## Data Availability

All study data are included in the article and/or supporting information.
