# Supplementary material for: RACK1A positively regulates opening of the apical hook in Arabidopsis thaliana via suppression of its auxin response gradient
Source: Proc Natl Acad Sci U S A. 2025 Jul 21;122(30):e2407224122. doi: 10.1073/pnas.2407224122 (PMC12318229; doi:10.1073/pnas.2407224122)
Supplement: Supplementary file 1 — Appendix 01 (PDF) [file pnas.2407224122.sapp.pdf]

# Supporting Information for

# RACK1A positively regulates opening of the apical hook in *Arabidopsis thaliana* via suppression of its auxin response gradient.

Qian Ma, Sijia Liu, Siamsa M. Doyle, Sara Raggi, Barbora Pařízková, Deepak Kumar Barange, Hemamshu Ratnakaram, Edward G. Wilkinson, Isidro Crespo Garcia, Joakim Bygdell, Gunnar Wingsle, Dirk Roeland Boer, Lucia C. Strader, Fredrik Almqvist, Ondřej Novák, and Stéphanie Robert.

Corresponding author: Stéphanie Robert
Email: stephanie.robert@slu.se

**This PDF file includes:**

Supporting text
Figures S1 to S8
Legend for Dataset S1
SI References

**Other supporting materials for this manuscript include the following:**

Dataset S1

## Supporting Information Text

### Detailed Materials and Methods

**Plant materials and growth conditions.** All *Arabidopsis thaliana* mutants and transgenic lines were in Columbia-0 (Col-0) ecotype background, except *shy2-2*, which was in Landsberg *erecta* (Ler) ecotype background. *Arabidopsis* lines *axr1-30* (1), *DR5::GUS* (2), *tir1-1afb2-3* (3), *axr2-1* (4), *shy2-2* (5), *arf2-8* and *arf7-1arf19-1* (6), *ARF7::ARF7-Venus* (7), *ARF19::ARF19-Venus* (8), *rack1a-3* and *amiR-rack1-es1* (9), *rack1b-2*, *rack1c-1* and *rack1b-2rack1c-1* (10), *RACK1A::RACK1A-GFP* in *rack1a-1* background (11) and *DR5::n3GFP* (12) were described previously. To generate *rack1a-3rack1b-2* and *rack1a-3rack1c-1* double mutants, genetic crossing was performed, and the homozygous lines were screened by PCR genotyping (no double homozygous line was obtained for *rack1a-3rack1b-2*). For experiments in which genotype combinations of *axr1-30* and *rack1a-3* were used (Fig. 4*C*, *D*, *E* and *SI Appendix*, Fig. S8*B*), they were assembled by genetic crossing and were simultaneously combined with *DR5::n3GFP*, as were all control lines including WT and single mutants. The homozygous lines were screened by fluorescence and PCR genotyping.

PCR genotyping was performed as previously described (13) using the following primers. For *rack1a-3* T-DNA mutant (SAIL_279_G02): WT allele, 5'-TTT CGG CAG ATG TAC GTA CTA C-3' and 5'-CTG AAT CTG ACG CAG CTA ACC-3'; T-DNA allele, 5'-TAG CAT CTG AAT TTC ATA ACC AAT CTC GAT ACA C-3' and 5'-CTG AAT CTG ACG CAG CTA ACC-3'. For *rack1b-2* T-DNA mutant (SALK_145920): WT allele, 5'- TCT CGA CCT CAA ACC CTG-3' and 5'-GAG AAG ACT TTA GAG TCG ATG GA-3'; T-DNA allele, 5'-ATT TTG CCG ATT TCG GAA C-3' and 5'-GAG AAG ACT TTA GAG TCG ATG GA-3'. For *rack1c-1* T-DNA mutant (SAIL_199_A04): WT allele, 5'-ACA ATA CTG ACG CAG TCT GG-3' and 5'-ATC TCT CGC TCT GTT ACG C-3'; T-DNA allele, 5'-TAG CAT CTG AAT TTC ATA ACC AAT CTC GAT ACA C-3' and 5'-ATC TCT CGC TCT GTT ACG C-3'. For *axr1-30* T-DNA mutant (SAIL_904_E06): WT allele, 5'-CAA ACC TGT TTT CTC TTC CAG G-3' and 5'-ATC TGT CCG CAG CTC TAA GAA G-3'; T-DNA allele, 5'-TAG CAT CTG AAT TTC ATA ACC AAT CTC GAT ACA C-3' and 5'-ATC TGT CCG CAG CTC TAA GAA G-3'.

For most experiments, surface-sterilized seeds were sown on solid half-strength Murashige and Skoog (MS) growth medium (2.2 g $L^{-1}$ MS medium with vitamins (Duchefa), 0.05% morpholinoethanesulfonic acid (Sigma-Aldrich), 1% w/v sucrose, 0.7% w/v plant agar (Duchefa), pH 5.6) supplemented with indicated concentrations of DAPIA, DAPIA metabolites and analogs, estradiol or equal volume of dimethyl sulfoxide (DMSO) solvent as the mock treatment, and incubated at 4ºC for two days for cold stratification. For ARF7 and ARF19 immunoblotting experiments, plant nutrient medium (14) with 0.5% w/v sucrose and 0.6% w/v agar was used. Assays were performed with etiolated seedlings; plates were incubated in white light at 22ºC for 6-12 h to stimulate seed germination and then vertically positioned in darkness at 22ºC. For hooks at the maintenance or late maintenance-opening phase, incubation in darkness was typically around 3 days (45-55 h after germination) or around 4 days (60-75 h after germination), respectively.

**Chemical screen.** The chemical screen was conducted as described previously (15). Briefly, a total number of 4,560 diverse compounds from ChemBridge were dissolved individually in DMSO as 5 mM stock solutions. In wells of 24-well plates, 300 µL solid growth medium was supplemented with compounds at 17 µM, with DMSO (mock) controls present in each plate. *Arabidopsis* seedlings of Col-0 and *axr1-30* were grown side by side within each well on the vertically positioned plates for 5 d under light conditions (white light for 16 h per day at 22ºC). In the first round, compounds affecting development in the WT, such as primary/lateral root growth, hypocotyl elongation and gravitropism response, but not or to a lesser extent in *axr1-30* seedlings, were selected. Their effects were confirmed in a second round of screening using fresh compound powders. DAPIA (ChemBridge ID 5327372) was selected from the reproducible hits as a compound of interest, which rescued the apical hook defect in 4-day-old *axr1-30* etiolated seedlings in a dose-dependent manner, without affecting the Col-0 apical hook phenotype at this time point.

**Chemical preparation.** Following the initial isolation of DAPIA from a screen of ChemBridge molecules (see Chemical screen above), we then synthesized DAPIA and the DAPIA analogs used for SAR analysis (see Synthetic procedures below; 16-18). DAPIA-N (3,5-dimethoxybenzamide) was purchased from TCI America (via Fisher Scientific). All other chemicals, including DAPIA-C (3,5-dimethoxybenzoic acid), were purchased from Sigma-Aldrich. Stock solutions of DAPIA, its metabolites and analogs, and estradiol, were prepared in DMSO.

**Kinematic analysis of apical hook development.** Seedlings were grown on vertically positioned solid medium plates at 22°C in a dark box illuminated with infrared light from 850 nm LEDs. Seedlings were photographed every hour, in order to catch the precise time of germination, for 10 days using a Canon D50 camera without an infrared filter. Hook angle was then quantified by the angle tool in ImageJ (Fiji) software every 3 or 4 h starting 12 h after germination. To analyze the effects of treatments or mutations on hook opening, the late maintenance-opening phase was considered as the section of a kinematic curve ranging from the time point at which the maximum mean hook angle was measured to that at which the mean hook angle first fell below 30% of its maximum. Statistical comparisons of mean hook opening rates were made using datasets of mean slope angles of the late maintenance-opening phase of each genotype/treatment to be compared. However, statistical comparisons of two growth curves were made at the same time points (the late maintenance-opening phase of the control or mock treatment, as specified in the figure legends).

**GUS staining.** Seedlings were grown on solid medium supplemented with chemicals as indicated for 4 d in darkness and then collected for GUS staining, which was performed as previously described (19). Briefly, seedlings were fixed in 80% acetone at -20°C for 20 min, washed with 0.1M phosphate buffer ($Na_2HPO_4$/$NaH_2PO_4$) three times, and transferred to GUS staining buffer (Na-phosphate buffer, 1 mg $mL^{-1}$ X-Gluc, 0.1% Triton X-100, 10 mM EDTA, 0.5 mM potassium ferrocyanide, and 0.5 mM potassium ferricyanide). Samples were incubated in darkness at 37°C for 9 h, followed by a series of clearing steps. Stained seedlings were then mounted on glass slides in a mixture of chloral hydrate:glycerol:$H_2O$ (8:3:1) and bright-field images were captured with a Leica DMi8 epifluorescence microscope.

**Confocal laser scanning microscopy and fluorescence quantification.** Fluorescence imaging was performed with Zeiss LSM 800 and 880 confocal microscopes. Images were acquired using identical imaging settings across samples within each experiment. For quantification of *ARF19::ARF19-Venus* and *DR5::n3GFP* fluorescence signal ratio across the apical hook, maximum intensity projections of z-stacks (with equal thickness and number of slices) were generated in ImageJ (Fiji) or ZEN (Zeiss) software and analyzed in ImageJ. The inner and outer hook sides were manually defined separately as regions of interest (ROIs) using the ImageJ polygon selection tool, starting directly after the bottom of the shoot apical meristem, and extending to an equal distance on the other side of the hook curvature (and using a similar ROI length for open hooks). For *ARF19::ARF19-Venus*, the mean gray value was measured in each ROI. For *DR5::n3GFP*, two images containing the fluorescent signal from either side were generated and the ImageJ plugin StarDist was then used with default settings to measure the nuclear fluorescence signal intensity as the mean gray value in each ROI. The fluorescence intensity ratio across the hook was expressed as inner:outer side ratio of fluorescence signal intensity. For *DR5::n3GFP*, this ratio represented the auxin response gradient across the hook. For the genotypes in which inner and outer sides could not be distinguished due to an open hook, the gradient was expressed as higher:lower fluorescence side ratio.

**Stability of DAPIA in medium and plants.** The stability of DAPIA was analyzed in both growth medium and plants. Col-0 and *axr1-30* seedlings were grown separately on solid growth medium supplemented with either DMSO or 10 μM DAPIA for 4 d in darkness. The media were collected directly after the solubilization of the chemicals (0 d) and after 4 d in the presence or absence of etiolated seedlings, at the same time point as which the seedlings were collected. All sample types were flash-frozen in liquid nitrogen and stored at -80°C until extraction. All experiments were done

in triplicate. An ACQUITY UPLC I-Class system combined with a Xevo TQ-S triple quadrupole mass spectrometer (Waters) was used to quantify DAPIA and its potential metabolites as previously described (20). Briefly, medium samples were heated in a microwave oven and then diluted 1/100 with methanol. For plant samples, 30 mg of tissue fresh weight was extracted in 100% methanol and purified by liquid-liquid extraction (MeOH:$H_2O$:hexane – 1:1:1). Each sample (1 μL) was then injected onto a reverse-phase column (Kinetex C18 100A, 50x2.1 mm, 1.7 μm; Phenomenex) and analyzed by the selected ion recording (SIR) modes for media samples ($[M+H]^+$: *m/z* 308, 182 and 183 for DAPIA, DAPIA-N and DAPIA-C, respectively) and multiple reaction monitoring (MRM) modes for plant tissue samples (308 > 165, 182 > 139, 183 > 77 for DAPIA, DAPIA-N and DAPIA-C, respectively). Quantification was performed by external calibration and the compounds were quantified according to their dilutions and/or estimated recoveries (24.2% for DAPIA, 67.7% for DAPIA-N and 45.3% for DAPIA-C). The limits of detection (signal-to-noise ratio of 1:3) were close to 0.1 pmol and 0.1 fmol for DAPIA and DAPIA-C, respectively, and 1 pmol or 10 fmol for DAPIA-N using SIR or MRM modes, respectively. The linear range was at least over 3 orders of magnitude with an $R^2$ coefficient of 0.997 to 0.999. All data were processed by MassLynx V4.1 software (Waters).

**ARF7-Venus and ARF19-Venus detection by immunoblotting.** Equal quantities of *ARF7::ARF7-Venus* and *ARF19::ARF19-Venus* 4-day-old etiolated seedlings for each tissue sample were collected under green light and flash frozen in liquid nitrogen. Tissue was homogenized using a 2010 Geno/Grinder (SPEX Sample Prep) at 1500 RPM for 30 sec and then placed at 70°C in 2x NuPage LDS buffer (141 mM Tris, 2% lithium dodecyl sulfide, 0.51 mM EDTA, 10% glycerol, 0.175 mM Phenol Red, 0.22 mM Coomassie blue) and loaded onto a Bolt 8% Bis-tris protein gel (Thermo Scientific). The gel was run at 80 V for 15 min and then 160 V for 1 h until proper separation of bands was obtained. The gel was then transferred to a nitrocellulose membrane (Amersham Protan 0.45 μm NC) using a wet-transfer system. The gel was washed with Ponceau solution (0.1% w/v Ponceau, 5% acetic acid) for 2 min, washed in TBS-T solution (200 mM Tris, 1.5 M NaCl, 0.1% Tween 20) until excess Ponceau solution was removed, and imaged for loading control analysis. The membrane was then blocked in 8% milk in TBS-T buffer for 1 h before incubation in rabbit anti-YFP antibody (Agrisera) at 1:5000 overnight at 4°C. The membrane was then washed 3 times for 5 min in TBS-T solution before being incubated with anti-rabbit HRP-coupled secondary antibody at 4°C overnight. The signal was then detected using a WesternBright ECL HRP substrate kit (Advasta) according to the manufacturer's instructions.

**DARTS assay.** The DARTS assay for target identification and validation of potential protein interactors of DAPIA was performed as described previously (21) with some modifications. For unbiased target identification, *Arabidopsis* 4-day-old etiolated seedlings were used for total protein extraction. All extraction steps were conducted at 4°C. After harvesting in green light, 0.5 g tissues were ground in liquid nitrogen, resuspended in 1 mL total protein extraction buffer (10 mM phosphate buffered saline (PBS) at pH 7.4 (Sigma-Aldrich), 0.5% v/v NP-40 (Nonidet™ P 40 Substitute from Sigma-Aldrich), 2 mM DTT, 1x protease inhibitors (Roche) and 1x phosphatase inhibitors (Roche) at a 1:2 w/v ratio), and centrifuged to discard the cell debris. After determining the protein concentration with Bradford protein assay reagents (Supelco via Merck), the protein extract at 5 mg $mL^{-1}$ was split into two LoBind 1.5 mL tubes and incubated with 100 μM DAPIA or equal volume of DMSO (1% v/v) as mock control for 1 h at room temperature with slow mixing. The compound concentration used was much higher than the biologically relevant dose in order to saturate the protein with ligand and ensure maximal protection from proteolysis (21). The treated protein extracts were further aliquoted, and each of the aliquots was mixed with pronase (Roche) (1.25 mg $mL^{-1}$ stock solution) at the dilution required to achieve the desired ratios of total enzyme to total protein substrate: 1:100, 1:300 and 0 (no pronase). After incubation for 30 min at room temperature, the proteolytic digestion was stopped by adding protease inhibitor cocktail (PIC) (Roche) and the tubes were placed on ice immediately. To prepare samples for proteomic analysis, all the following steps were performed on-column using Vivacon 500 10K Spin Columns (Sartorius Stedim). The DARTS protein samples were denatured and reduced in denaturing buffer (6 M

guanidine, 0.1 M Tris and 5 mM EDTA, pH 8.0) containing 0.3% w/v DTT at 70°C for 1 h, alkylated in alkylation buffer (1.5% w/v iodoacetamide in denaturing buffer) at room temperature for 30 min in the dark, and digested by trypsin (Promega) at 1:100 w:w enzyme-to-sample ratio for each sample in 50 mM ammonium bicarbonate, pH 8.0, at 37°C for 16 h in the dark with gentle rotation. After column centrifugation, the flow-through was collected, containing the tryptic peptides to be analyzed by LC-MS/MS, leading to the identification of RACK1A as a potential target of DAPIA.

**LC−MS/MS analysis of tryptic peptides.** A 1 µg aliquot of each trypsin digested sample was loaded on a BEH C18 analytical column (75 µm internal diameter × 250 mm, 1.7 µm particles; Waters) and separated using a concave 180 min gradient of 1–40% solvent B (0.1% formic acid in acetonitrile) in solvent A (0.1% aqueous formic acid) at a flow rate of 368 nL $min^{-1}$. The eluate was passed to a nano-electrospray ionization-equipped Synapt G2-Si HDMS mass spectrometer (Waters) operating in a resolution mode. All data were collected using ion-mobility-$MS^E$ with dynamic range extension enabled using a scan time of 0.4 s, mass-corrected using Glu-fibrinopeptide B and Leu-enkephalin as reference peptides. The LC-MS/MS data were processed with Protein Lynx Global Server v.3.0.3 (Waters), and the resulting spectra were searched against the *Arabidopsis* TAIR10 database. The database search settings were: enzyme-specific cleavage with one miscleavage allowed; carbamidomethylated cysteines as fixed modification; oxidized methionine, N-terminal acetylation, and deamidated asparagine and glutamine as variable modifications. A minimum of three fragments were required for peptide detection with a precursor and fragment tolerance of 10 and 25 ppm, respectively, with a false discovery rate < 5%.

**RACK1A detection by immunoblotting.** For validation of ligand binding to its potential target, compound-treated protein extracts were divided into six aliquots of 50 µL, to which different dilutions of pronase were added as indicated and digested for 30 min at room temperature. After mixing with 4x Laemmli sample buffer (Bio-Rad) containing 2-mercaptoethanol and boiling at 70°C for 10 min to stop the proteolytic reaction, the protein samples were loaded onto SDS-PAGE gels and immunoblotting was performed. Membranes were probed with either rabbit anti-RACK1A (1:500) (22) or mouse anti-α-Tubulin (1:3000; Sigma-Aldrich) antibodies. The secondary antibodies were HRP-coupled goat anti-rabbit (Agrisera) and goat anti-mouse (Santa Cruz). Blots were developed with SuperSignal West Dura Extended Duration Substrate (Thermo Scientific) according to the manufacturer's instructions, or stained with Coomassie blue, and imaged with a Bio-Rad ChemiDoc XRS+ molecular imager.

**Molecular docking.** The 3D structure of the DAPIA ligand was generated with Marvin 18.24.0 (Chemaxon). The PDB-entry 3DM0 contained the crystal structure of *Arabidopsis* RACK1A as a C-terminal fusion with maltose binding protein (MBP). A pdb file containing only the RACK1A structure, encompassing residues Leu5 to Ile324 of the protein's 327 amino acids, was generated with the open-source software PyMOL (version 1.7, Schrödinger) by removing the MBP sequence and used for computational docking. AutoDockTools version 1.5.6 (23) was used for pdbqt-format preparation of the protein and ligand. Docking simulations were executed with AutoDock 4.2 (24). A blind docking encompassing the full protein structure was first performed to predict one or more optimized poses for the ligand, followed by local docking focusing on the optimized pose areas to find the most favorable pose. The default parameters in AutoDock that are suitable for most drug-sized ligands were used, except 'ga_pop_size' and 'ga_run', which were set to 300 and 500, respectively. For blind dockings, the grid-box size was x, y and z = 126 with Grid Point Spacing = 0.453 Å centered at x = 54.959, y = 34.163, and z = 31.695, while x, y = 70 and z = 112 with 0.375 Å centered at x = 54.959, y = 36.284, and z = 31.695 for local dockings. The binding site was predicted by the conformational cluster with the lowest estimated free energy of binding and the highest number of runs. The pose of the ligand with the lowest estimated free energy within the binding site was selected and displayed graphically. UCSF Chimera (25) was used for visualization.

**Microscale thermophoresis (MST) analysis.** Purified insect cell-expressed RACK1A was fluorescently labeled with Protein Labeling Kit RED-NHS 2nd Generation (NanoTemper

Technologies GmbH) via amine conjugation. Increasing concentrations of titrant (DAPIA or its inactive analogs) were titrated against constant concentrations (30 nM) of the labelled RACK1A protein in a standard MST buffer (50 mM Tris, pH 7.5, 150 mM NaCl, 10 mM $MgCl_2$, 0.05% Tween 20). DAPIA and its inactive analogs DAPIA-02 and DAPIA-07 were dissolved in DMSO for a final concentration of 5% v/v when added to an equal volume of target protein solution. MST premium-coated capillaries (Monolith) were used to load the samples into a Monolith NT.115 MST instrument (NanoTemper Technologies GmbH). The LED power and MST power were set at 60% and 40%, respectively, for all the thermophoresis measurements. Before the quantitative binding affinity assays, a binding check procedure was performed to analyze whether the experiment set-up was correct and a binding event could be detected (yes/no answer). MST experiments were performed at room temperature and standard deviation was calculated from four independent replicates. Data were analyzed using the MO.Affinity Analysis 3 software provided together with the instrument and the dissociation constant ($K_d$) was calculated by the $K_d$ fitting function of the software.

**RT-qPCR.** Approximately the upper third of the shoots of around 20 etiolated seedlings grown for 4 days in darkness (including germination time) were rapidly removed with a scalpel and pooled under green light before flash freezing in liquid nitrogen and grinding with a bead mill. This was repeated for a total of three independent biological replicates on different days. Total RNA was extracted from each sample using the Plant RNeasy Kit (Qiagen) according to the manufacturer's instructions. RQ1 RNase-free DNase (Promega) was used for the on-column DNase digestion step. RNA concentration was measured with a NanoDrop 2000 spectrophotometer (Thermo Fisher Scientific). cDNA was prepared from 1 µg total RNA with the iScript cDNA Synthesis Kit (Bio-Rad), following the manufacturer's instructions. Serial dilutions of pooled cDNA from all samples for a particular experiment were used to determine efficiencies for each primer pair. RT-qPCR analyses on two technical replicates per sample were performed on a CFX96 Touch System (Bio-Rad) using SsoAdvanced Universal SYBR Green Supermix (Bio-Rad), an mRNA amplification protocol of 95°C for 3 min followed by 46 cycles of 95°C for 10 sec and 60°C for 30 sec, and the following primers: 5'-GCT GAA AAG GCT GAC AAC AGT-3' and 5'-GCT CCA GTT AAG GCT TGT GC-3' for *RACK1A* (AT1G18080), 5'-TTG TTG AGG ATT TGA AGG TTG A-3' and 5'-CCA GTT CAA GCT TGT GCA GTA-3' for *RACK1B* (AT1G48630), and 5'-GAG GCA GAG AAG AAT GAA GGT G-3' and 5'-CCA GTT CAA GCT TGT GCA GTA-3' for *RACK1C* (AT3G18130) (9). RT-qPCR analyses of the two stable reference genes *PEX4* (AT5G25760) and *PP2AA3* (AT1G13320) were also performed on the samples, for which the primers used were 5'-CTT AAC TGC GAC TCA GGG AAT CTT C-3' and 5'-AGG CGT GTA TAC ATT TGT GCC ATT-3', and 5'-TAA CGT GGC CAA AAT GAT GC-3' and 5'-GTT CTC CAC AAC CGC TTG GT-3', respectively (26). Expression levels of the target genes were calculated as described previously (26) and *RACK1* gene expression levels were then normalized to that of the two reference genes by geometric averaging (27). For each target gene, the normalized expression values were then scaled relative to that of the WT control.

**Statistical analysis.** For statistical analysis of differences among multiple sample groups, one-way ANOVA and Tukey's multiple comparison tests were performed and different letters in the figures represent significant differences at $P < 0.05$. For statistical analysis of differences between two sample groups, the Student's T-test or Wilcoxon rank sum test was performed for parametric or non-parametric datasets, respectively. The Compare Groups of Growth Curves (CGGC) method, which performs permutation tests to compare curves of measurements over time (28), was used to statistically analyze differences between apical hook angle kinematic curves. This method calculates all pairwise comparisons with T-tests at each time point and averages them to obtain the permutation *P*-value (28). Statistical differences are represented as follows in the figures: different letters – $P < 0.05$; ns – not significantly different; *$P < 0.05$; **$P < 0.01$; ***$P < 0.001$. Error bars represent either standard deviation (SD) or standard error of the mean (SEM), as indicated in the figure legends. Biological replicates were performed on different days. Line graphs, bar graphs and box plots were drawn using Excel (Microsoft), Prism (GraphPad) and Origin (OriginLab) software.

**Synthetic procedures for DAPIA, DAPIA-01, DAPIA-02, DAPIA-03, DAPIA-05, DAPIA-06, DAPIA-07, DAPIA-08, DAPIA-09 and DAPIA-10.**

DAPIA-C (CAS: 1132-21-4) and DAPIA-N (CAS: 17213-58-0) were purchased.

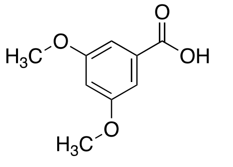


3,5-dimethoxybenzoic acid

DAPIA-C

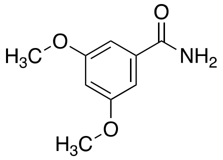


3,5-dimethoxybenzamide

DAPIA-N

DAPIA-C was used as a precursor to synthesize DAPIA-01 and DAPIA-02. DAPIA-02 was used to synthesize DAPIA-03 and DAPIA-09. DAPIA-05 was used to synthesize DAPIA-08 and DAPIA-08 was used to synthesize DAPIA-10.

**General Experimental procedure for DAPIA, DAPIA-01, DAPIA-02, DAPIA-05, DAPIA-06 and DAPIA-07.** A two neck, round-bottomed flask fitted with reflux condenser and equipped with a magnetic stirring bar charged substituted carboxylic acid (100-500 mg scale) and $CH_2Cl_2$ (20-100 mL). The flask was closed with a rubber septum and placed under nitrogen by piercing the septum with a needle. Oxalyl chloride (2 equiv.) was added dropwise via a syringe through the septum followed by the addition of a few drops of DMF. The resulting mixture was refluxed for 6 h (monitored by TLC) and then concentrated on a rotary evaporator. To the resulting crude acid chloride, THF (10-50 mL) was added. To this solution, diisopropylethylamine (DIPEA) (2 equiv.) was added and the mixture was cooled in an ice bath to 0°C and stirred at the same temperature for 30 min. To this mixture, substituted amine (1.5 equiv.) was added and the reaction mixture was stirred at room temperature for 12 h. The mixture was treated with 2 N HCl (adjusted pH to 7), transferred to a separatory funnel and extracted with ethyl acetate. The organic layers were washed with brine, dried over sodium sulfate, and concentrated under reduced pressure to give crude material, which was purified by crystallization (ethyl acetate and hexane) or trituration with hexane to afford the desired product (48 to 80%).

**Synthesis of DAPIA:**

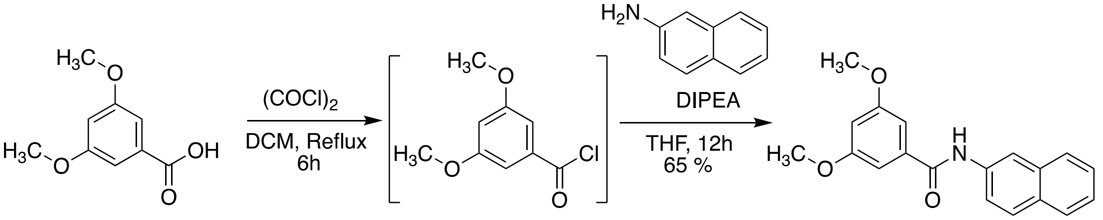


**Experimental procedure for DAPIA.** A two neck, 25 mL round-bottomed flask equipped with a magnetic stirring bar charged 3,5-dimethoxybenzoic acid (50 mg, 0.274 mmol) and $CH_2Cl_2$ (10 mL). The flask was closed with a rubber septum and placed under nitrogen by piercing the septum with a needle. Oxalyl chloride (0.047 mL, 0.069 g, 0.549 mmol) was added dropwise via a syringe through the septum followed by the addition of 1 drop of DMF. The resulting mixture was refluxed for 6 h (monitored by TLC) and then concentrated on a rotary evaporator. To the resulting crude acid chloride, THF (10 mL) was added. To this solution, diisopropylethylamine (DIPEA) (0.095 mL, 0.071 g, 0.549 mmol) was added and the mixture was cooled in an ice bath to 0°C and stirred at the same temperature for 30 min. To this mixture, anthracene-2-amine (0.05 g, 0.274 mmol) was added, and the reaction mixture was stirred at room temperature for 12 h. The mixture was treated with 2 N HCl (adjusted pH to 7), transferred to a 50 mL separatory funnel and extracted with ethyl acetate (25 mL x 3). The organic layers were washed with brine (25 mL), dried over sodium sulfate,

concentrated under reduced pressure and triturated with hexane to afford DAPIA as an off-white solid (55 mg, 65%).

**Synthesis of DAPIA-01:**

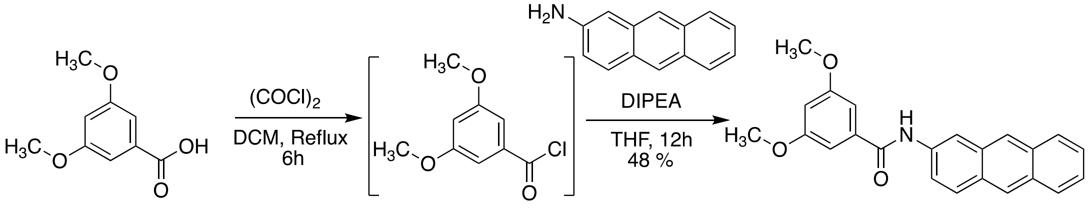


**Experimental procedure for DAPIA-01.** A two neck, 50 mL round-bottomed flask equipped with a magnetic stirring bar charged 3,5-dimethoxybenzoic acid (100 mg, 0.548 mmol) and $CH_2Cl_2$ (20 mL). The flask was closed with a rubber septum and placed under nitrogen by piercing the septum with a needle. Oxalyl chloride (0.094 mL, 0.139 g, 1.09 mmol) was added dropwise via a syringe through the septum followed by the addition of 1 drop of DMF. The resulting mixture was refluxed for 6 h (monitored by TLC) and then concentrated on a rotary evaporator. To the resulting crude acid chloride, THF (20 mL) was added. To this solution, diisopropylethylamine (DIPEA) (0.191 mL, 0.142 g, 1.09 mmol) was added and the mixture was cooled in an ice bath to 0°C and stirred at the same temperature for 30 min. To this mixture, anthracene-2-amine (0.159 g, 0.823 mmol) was added, and the reaction mixture was stirred at room temperature for 12 h. The mixture was treated with 2 N HCl (adjusted pH to 7), transferred to a 100 mL separatory funnel and extracted with ethyl acetate (50 mL x 3). The organic layers were washed with brine (100 mL), dried over sodium sulfate, concentrated under reduced pressure, and triturated with hexane to afford DAPIA-01 as an off-white solid (95 mg, 48%).

**Synthesis of DAPIA-02:**

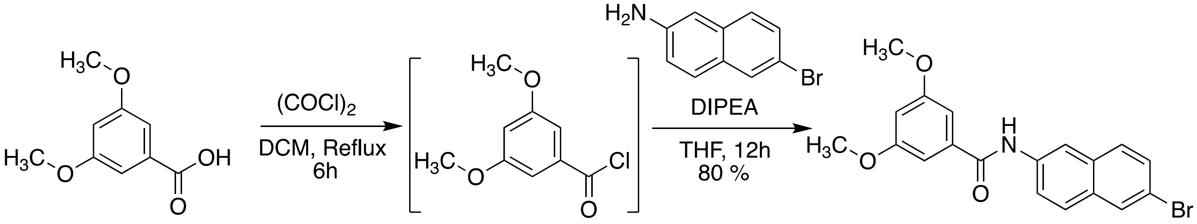


**Experimental procedure for DAPIA-02.** A two neck, 250 mL round-bottomed flask equipped with a magnetic stirring bar charged 3,5-dimethoxybenzoic acid (500 mg, 2.74 mmol) and $CH_2Cl_2$ (100 mL). The flask was closed with a rubber septum and placed under nitrogen by piercing the septum with a needle. Oxalyl chloride (0.47 mL, 0.696 g, 5.48 mmol) was added dropwise via a syringe through the septum followed by the addition of 2 drops of DMF. The resulting mixture was refluxed for 6 h (monitored by TLC) and then concentrated on a rotary evaporator. THF was added to the resulting crude acid chloride. To this solution, diisopropylethylamine (DIPEA) (0.949 mL, 0.709 g, 5.48 mmol) was added and the mixture was cooled in an ice bath to 0°C and stirred at the same temperature for 30 min. To this mixture, 6-bromonaphthalen-2-amine (0.914 g, 4.11 mmol) was added and the reaction mixture was stirred at room temperature for 12 h. The mixture was treated with 2 N HCl (adjusted pH to 7), transferred to a 100 mL separatory funnel and extracted with ethyl acetate (50 mL x 3). The organic layers were washed with brine (100 mL) and dried over sodium sulfate, concentrated under reduced pressure, and triturated with hexane to afford DAPIA-02 as an off-white solid (0.85 g, 80%).

**Synthesis of DAPIA-03:**

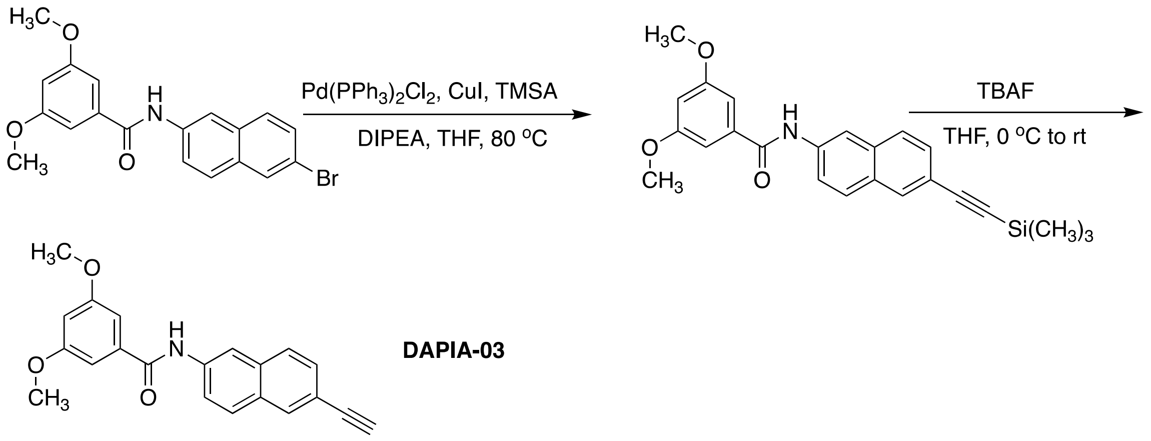


**Experimental procedure for DAPIA-03.** A mixture of *N*-(6-bromonaphthalen-2-yl)-3,5-dimethoxybenzamide (100 mg, 0.25 mmol), $Pd(PPh_3)Cl_2$ (18.17 mg, 0.025 mmol), CuI (3.94 mg, 0.02 mmol), and diisopropylethylamine (DIPEA) (0.103 g, 0.134 mL, 0.776 mmol) in THF (20 mL) was stirred at 25°C for 30 min under nitrogen. Trimethylsilyl acetylene (TMSA) (0.107 mL, 76.2 mg, 0.776 mmol) was added slowly to the mixture with stirring. The resulting mixture was then stirred at 25°C for 12 h, diluted with water (50 mL), and extracted with ethyl acetate (100 mL x 3). The organic layers were collected, combined, dried over anhydrous $Na_2SO_4$, and concentrated under reduced pressure. The residue was then purified by column chromatography using hexane-ethyl acetate to give the desired product as a low melting solid (80 mg, 76% yield). Sonogashira coupling product was used for the next step for TMS-deprotection. The coupled product (50 mg, 0.123 mmol) was taken in dry THF (10 mL) and cooled in an ice bath. TBAF (1M in THF, 0.148 mL, 0.148 mmol) was added and the reaction mixture was allowed to warm to RT and stirred for 12 h. The mixture was concentrated *in vacuo*, diluted with ethyl acetate and washed with sat aq. $NH_4Cl$. The organic layer was concentrated to afford crude material, which was purified by column chromatography using ethyl acetate:hexane to afford an off-white solid (20 mg, 48%).

**Synthesis of DAPIA-05:**

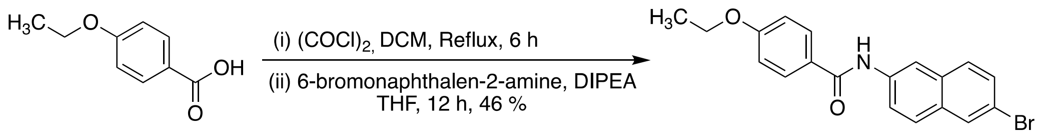


**Experimental procedure for DAPIA-05.** A two neck, 100 mL round-bottomed flask equipped with a magnetic stirring bar charged 4-ethoxybenzoic acid (200 mg, 1.2 mmol) and $CH_2Cl_2$ (30 mL). The flask was closed with a rubber septum and placed under nitrogen by piercing the septum with a needle. Oxalyl chloride (0.206 mL, 305.5 g, 2.4 mmol) was added via a syringe through the septum followed by the addition of 2 drops of DMF. The resulting mixture was refluxed for 6 h (monitored by TLC) and then concentrated on a rotary evaporator. To the resulting crude yellow acid chloride, $CH_2Cl_2$ (10 mL) was added. To this solution, diisopropylethylamine (DIPEA) (0.416 mL, 0.311 g, 2.4 mmol) was added and the mixture was cooled in an ice bath to 0°C and stirred at the same temperature for 30 min. To this mixture, 6-bromonaphthalen-2-amine (0.400 g, 1.8 mmol) was added, and the reaction mixture was stirred at room temperature for 12 h. The mixture was treated with 2 $NH_4Cl$ (adjusted pH to 7), transferred to a 100 mL separatory funnel and extracted with ethyl acetate (50 mL x 3). The organic layers were washed with brine (30 mL) and then concentrated under reduced pressure and triturated to afford DAPIA-05 as a white solid (0.205 g, 46%).

**Synthesis of DAPIA-06:**

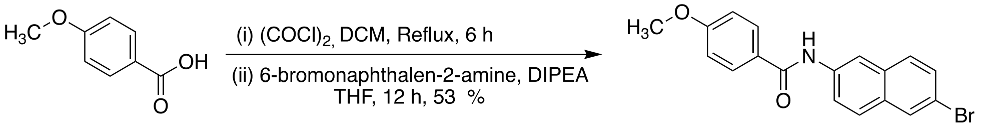


DAPIA-06

**Experimental procedure for DAPIA-06.** A two neck, 50 mL round-bottomed flask equipped with a magnetic stirring bar charged 4-methoxybenzoic acid (100 mg, 0.678 mmol) and $CH_2Cl_2$ (20 mL). The flask was closed with a rubber septum and placed under nitrogen by piercing the septum with a needle. Oxalyl chloride (0.113 mL, 0.167 g, 1.31 mmol) was added via a syringe through the septum followed by the addition of 1 drop of DMF. The resulting mixture was refluxed for 6 h (monitored by TLC) and then concentrated on a rotary evaporator. To the resulting crude yellow acid chloride, $CH_2Cl_2$ (10 mL) was added. To this solution, diisopropylethylamine (DIPEA) (0.228 mL, 0.17 g, 1.31 mmol) was added and the mixture was cooled in an ice bath to 0°C and stirred at the same temperature for 30 min. To this mixture, 6-bromonaphthalen-2-amine (0.218 g, 0.986 mmol) was added and the reaction mixture was stirred at room temperature for 12 h. The mixture was treated with 2 N HCl (adjusted pH to 7), transferred to a 100 mL separatory funnel and extracted with ethyl acetate (50 mL x 3). The organic layers were washed with brine (30 mL) and then concentrated under reduced pressure and triturated to afford DAPIA-06 as a white solid (0.125 g, 53%).

**Synthesis of DAPIA-07:**

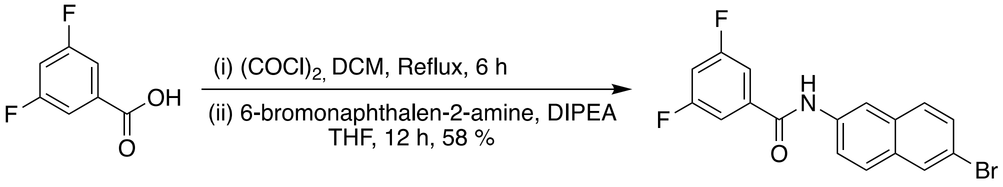


DAPIA-07

**Experimental procedure for DAPIA-07.** A two neck, 50 mL round-bottomed flask equipped with a magnetic stirring bar charged 4-ethoxybenzoic acid (100 mg, 0.632 mmol) and $CH_2Cl_2$ (20 mL). The flask was closed with a rubber septum and placed under nitrogen by piercing the septum with a needle. Oxalyl chloride (0.108 mL, 0.161 g, 1.26 mmol) was added via a syringe through the septum followed by the addition of 1 drop of DMF. The resulting mixture was refluxed for 6 h (monitored by TLC) and then concentrated on a rotary evaporator. To the resulting crude acid chloride, $CH_2Cl_2$ (10 mL) was added. To this solution, diisopropylethylamine (DIPEA) (0.219 mL, 0.163 g, 1.26 mmol) was added and the mixture was cooled in an ice bath to 0°C and stirred at the same temperature for 30 min. To this mixture, 6-bromonaphthalen-2-amine (0.211 g, 0.948 mmol) was added, and the reaction mixture was stirred at room temperature for 12 h. The mixture was treated with 2 N HCl (adjusted pH to 7), transferred to a 100 mL separatory funnel and extracted with ethyl acetate (50 mL x 3). The organic layers were washed with brine (30 mL) and then concentrated under reduced pressure and triturated to afford DAPIA-07 as a white solid (0.134 g, 58%).

**Synthesis of DAPIA-08:**

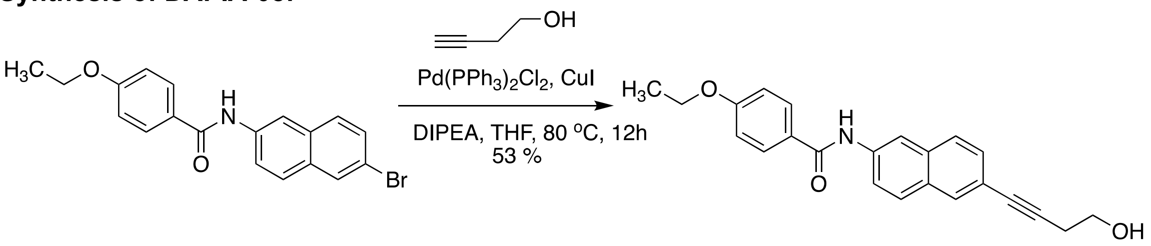


**Experimental procedure for DAPIA-08.** A mixture of *N*-(6-bromonaphthalen-2-yl)-4-ethoxybenzamide (0.2 g, 0.54 mmol), $Pd(PPh_3)Cl_2$ (0.038 g, 0.054 mmol), CuI (0.0083 mg, 0.043 mmol) and diisopropyl ethylamine (DIPEA) (0.28 g, 0.209 mL, 1.62 mmol) in THF (8 mL) was stirred at 25°C for 30 min under nitrogen. 3-Butyn-ol (0.122 mL, 113.9 mg, 1.62 mmol) was added slowly to the mixture with stirring. The resulting mixture was then heated at 80°C for 12 h, diluted with water (50 mL), and extracted with ethyl acetate (50 mL x 3). The organic layers were collected, combined, dried over anhydrous $Na_2SO_4$ and concentrated under reduced pressure. The residue was then purified by column chromatography using hexane-ethyl acetate to give DAPIA-08 (98 mg, 53%).

**Synthesis of DAPIA-09:**

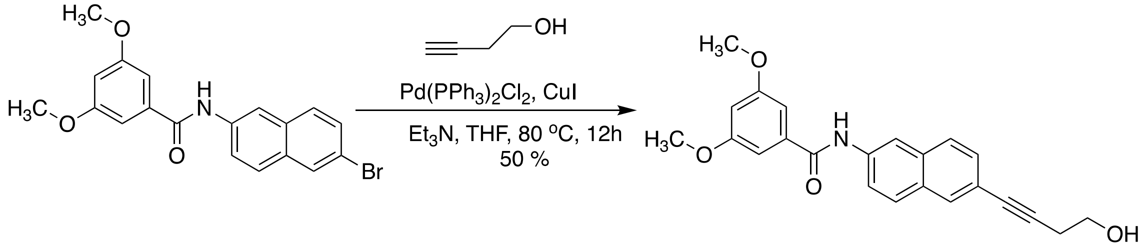


**Experimental procedure for DAPIA-09.** A mixture of *N*-(6-bromonaphthalen-2-yl)-3,5-dimethoxybenzamide (0.1 g, 0.258 mmol), $Pd(PPh_3)Cl_2$ (0.018 mg, 0.0259 mmol), CuI (0.0039 g, 0.0207 mmol) and diisopropylethylamine (DIPEA) (0.134 mL, 0.1 g, 0.776 mmol) in THF (8 mL) was stirred at 25°C for 30 min under nitrogen. 3-Butyn-1-ol (0.058 mL, 0.054 g, 0.778 mmol) was added slowly to the mixture with stirring. The resulting mixture was then heated at 80°C for 12 h, diluted with water (20 mL), and extracted with ethyl acetate (25 mL x 3). The organic layers were collected, combined, dried over anhydrous $Na_2SO_4$ and concentrated under reduced pressure. The residue was then purified by column chromatography using hexane-ethyl acetate to afford DAPIA-09 as an off-white solid (50 mg, 50%).

**Synthesis of DAPIA-10:**

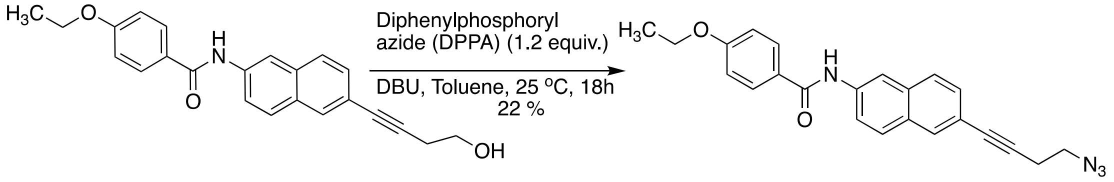


**Experimental procedure for DAPIA-10.** Diphenylphosphoryl azide (0.146 mL, 0.179.9 g, 0.653 mmol) and DBU (0.117 mL, 0.119 g, 0.789 mmol) were added to a suspension of 4-ethoxy-*N*-(6-(4-hydroxybut-1-yn-1-yl)naphthalen-2-yl)benzamide (90 mg, 0.261 mmol) in toluene (50 mL) at 25°C under nitrogen. The resulting mixture was stirred at 25°C for 18 h. The reaction mixture was quenched with sat aq. $NaHCO_3$ (10 mL). The reaction mixture was extracted with EtOAc (150 mL).

The combined organics were dried over sodium sulfate and concentrated to a yellow gum. The crude material was purified by column chromatography using ethyl acetate:hexane to provide the product, DAPIA-10, as an off-white solid (22 mg, 22%).

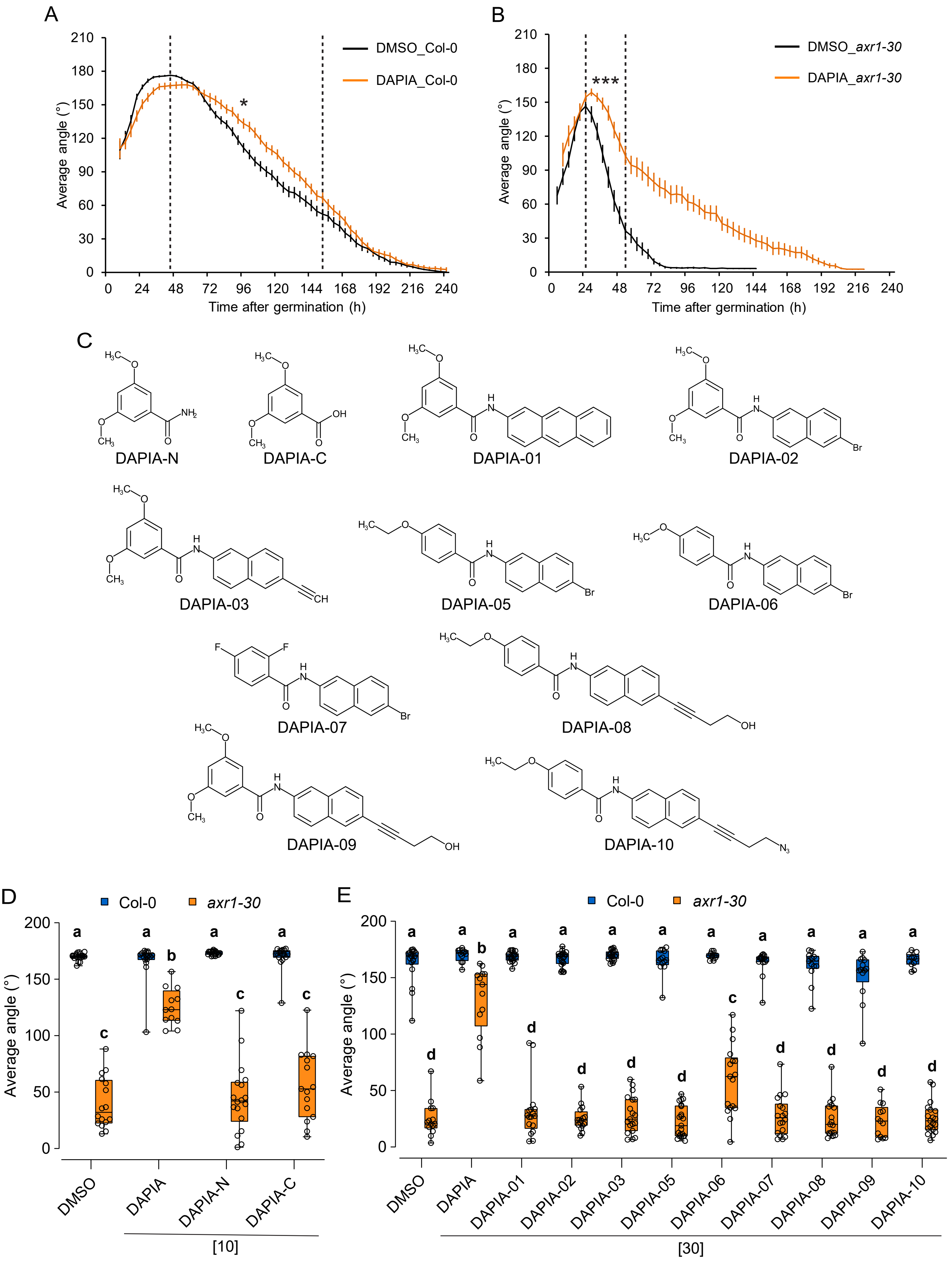
A
DMSO_Col-0
DAPIA_Col-0
Average angle (°)
Time after germination (h)
B
DMSO_axr1-30
DAPIA_axr1-30
***
C
DAPIA-N
DAPIA-C
DAPIA-01
DAPIA-02
DAPIA-03
DAPIA-05
DAPIA-06
DAPIA-07
DAPIA-08
DAPIA-09
DAPIA-10
D
Col-0
axr1-30
DMSO
DAPIA
[10]
E
[30]

**Figure S1.** DAPIA-induced deceleration of apical hook opening and DAPIA structure-activity relationship (SAR). (*A* and *B*) Kinematics of apical hook angle in Col-0 (*A*) and *axr1-30* (*B*) as measured every 4 h for 10 d of growth starting from germination (0 h) in darkness on medium supplemented with DMSO (mock) or 10 µM DAPIA. Error bars represent SEM; $N$ = 23-61 seedlings. Dashed lines indicate the late maintenance-opening phase (from the maximum mean hook angle to the first mean hook angle below 30% of the maximum) of the mock-treated control, for which asterisks indicate significantly different kinematic curves (CGGC method; *$P < 0.05$; ***$P < 0.001$). (*C*) Chemical structures of potential metabolites and analogs of DAPIA. (*D* and *E*) DAPIA SAR analysis – quantification of average hook angle after 4 d of growth (including germination time) of Col-0 and *axr1-30* in darkness on medium supplemented with DMSO (mock), DAPIA and its potential metabolites (*D*) or analogs (*E*). Values in square brackets represent concentrations in µM. Data is represented as box plots and different letters indicate significantly different means of $N$ = 11-21 seedlings at $P < 0.05$ (one-way ANOVA, Tukey's multiple comparison test).

A **Medium**

| | DAPIA [µM] | DAPIA-N [µM] | DAPIA-C [µM] |
|---|---|---|---|
| 0 d no plants | 9.29 ± 1.54 | nd | nd |
| 4 d no plants | 9.90 ± 2.36 | nd | nd |
| 4 d Col-0 | 5.17 ± 0.62 | nd | nd |
| 4 d *axr1-30* | 4.36 ± 0.09 | nd | nd |

**Plants**

| | DAPIA (pmol/mg FW) | DAPIA-N (pmol/mg FW) | DAPIA -N (%) | DAPIA-C (pmol/mg FW) |
|---|---|---|---|---|
| 4 d Col-0 | 472.83 ± 23.12 | 0.021 ± 0.002 | 0.004 | nd |
| 4 d *axr1-30* | 476.04 ± 30.90 | 0.022 ±0.002 | 0.005 | nd |

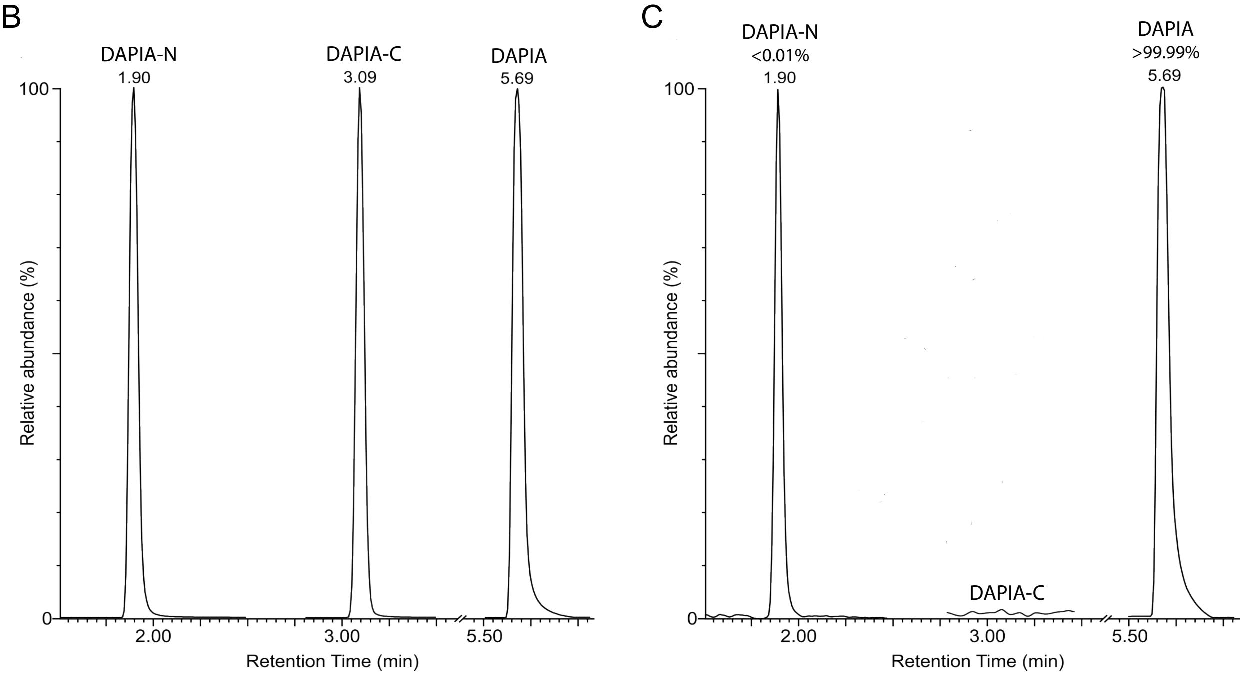


**Figure S2.** Stability of DAPIA *in vitro* and *in planta*. (*A*) Growth medium was supplemented with 10 µM DAPIA and Col-0 and *axr1-30* seedlings were then germinated and grown on the medium for 4 d in darkness. DAPIA, DAPIA-N and DAPIA-C concentrations were analyzed in the medium directly upon addition of DAPIA (0 d) and 4 d later (4 d) in the absence or presence of seedlings grown on the medium (Medium) and in the seedlings after the 4 d of growth (Plants). ± values represent SD; nd – not detectable. (*B*) Chromatographic separation of DAPIA (retention time 5.69 min, MRM 308 > 165) and its potential degradation products, DAPIA-N (retention time 1.90 min, MRM 182 > 139) and DAPIA-C (retention time 3.09 min, MRM 183 > 77). (*C*) Representative multi-MRM chromatograms of plant extract. Col-0 and *axr1-30* seedlings were germinated and grown for 4 d in darkness on medium supplemented with 10 µM DAPIA. After sample extraction, identification and quantification of analytes were performed using an LC-MS/MS system. DAPIA-N was detected at levels corresponding to less than 0.01% of total DAPIA concentration, while DAPIA-C was not detectable, in the plants. All experiments were done in triplicate.

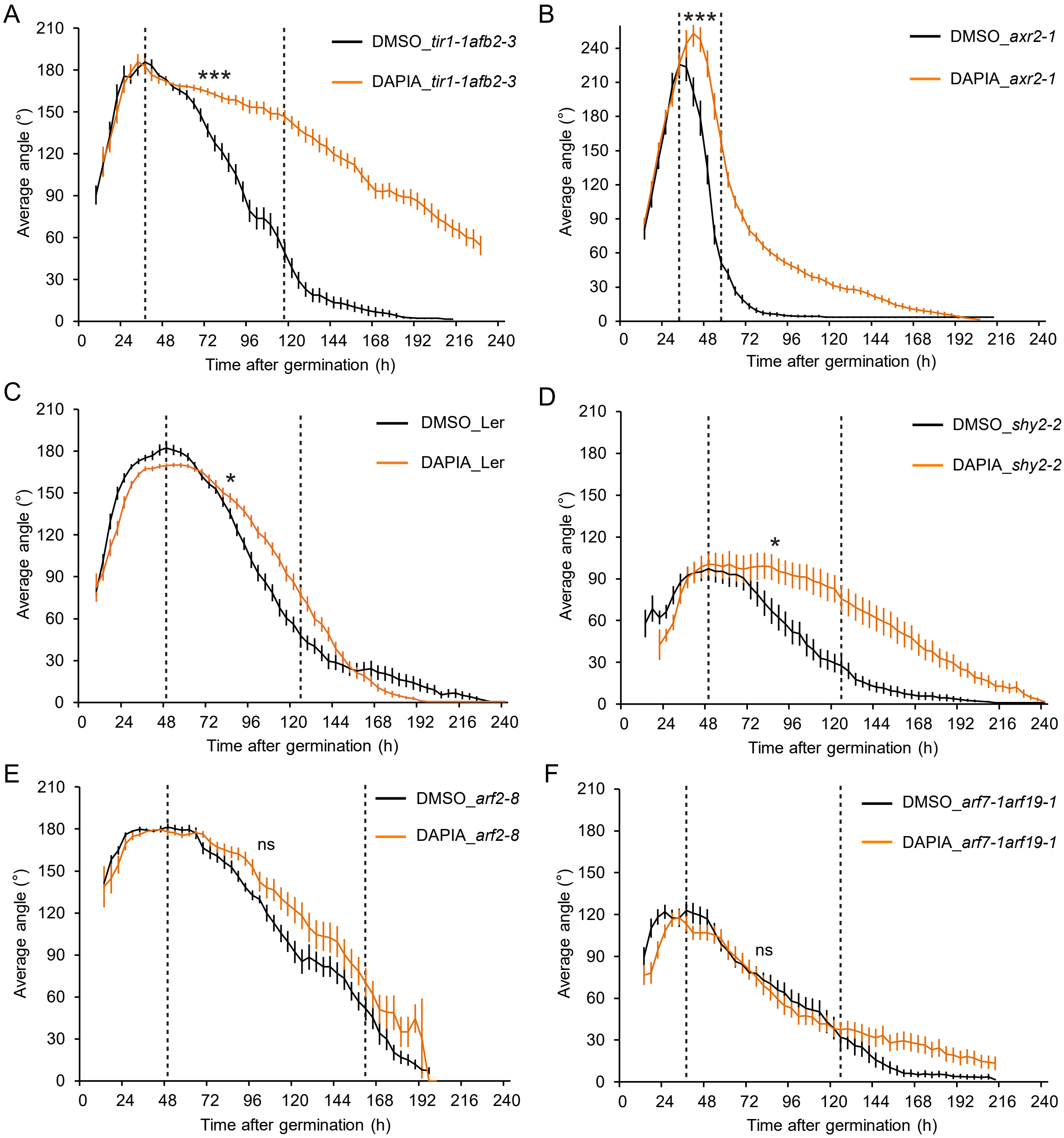


**Figure S3.** DAPIA requires the auxin signaling components AXR2, ARF7 and ARF19 to decelerate hook opening. (*A* to *F*) Kinematics of apical hook angle in *tir1-1afb2-3* (*A*), *axr2-1* (*B*), the Ler WT (*C*), *shy2-2* (*D*), *arf2-8* (*E*) and *arf7-1arf19-1* (*F*), as measured every 4 h for 10 d of growth starting from germination (0 h) in darkness on medium supplemented with DMSO (mock) or 10 µM DAPIA. Error bars represent SEM; *N* = 16-38 seedlings. Dashed lines indicate the late maintenance-opening phase (from the maximum mean hook angle to the first mean hook angle below 30% of the maximum) of the mock-treated control, for which asterisks indicate significantly different kinematic curves (CGGC method; ns – not significantly different; *P < 0.05; ***P < 0.001).

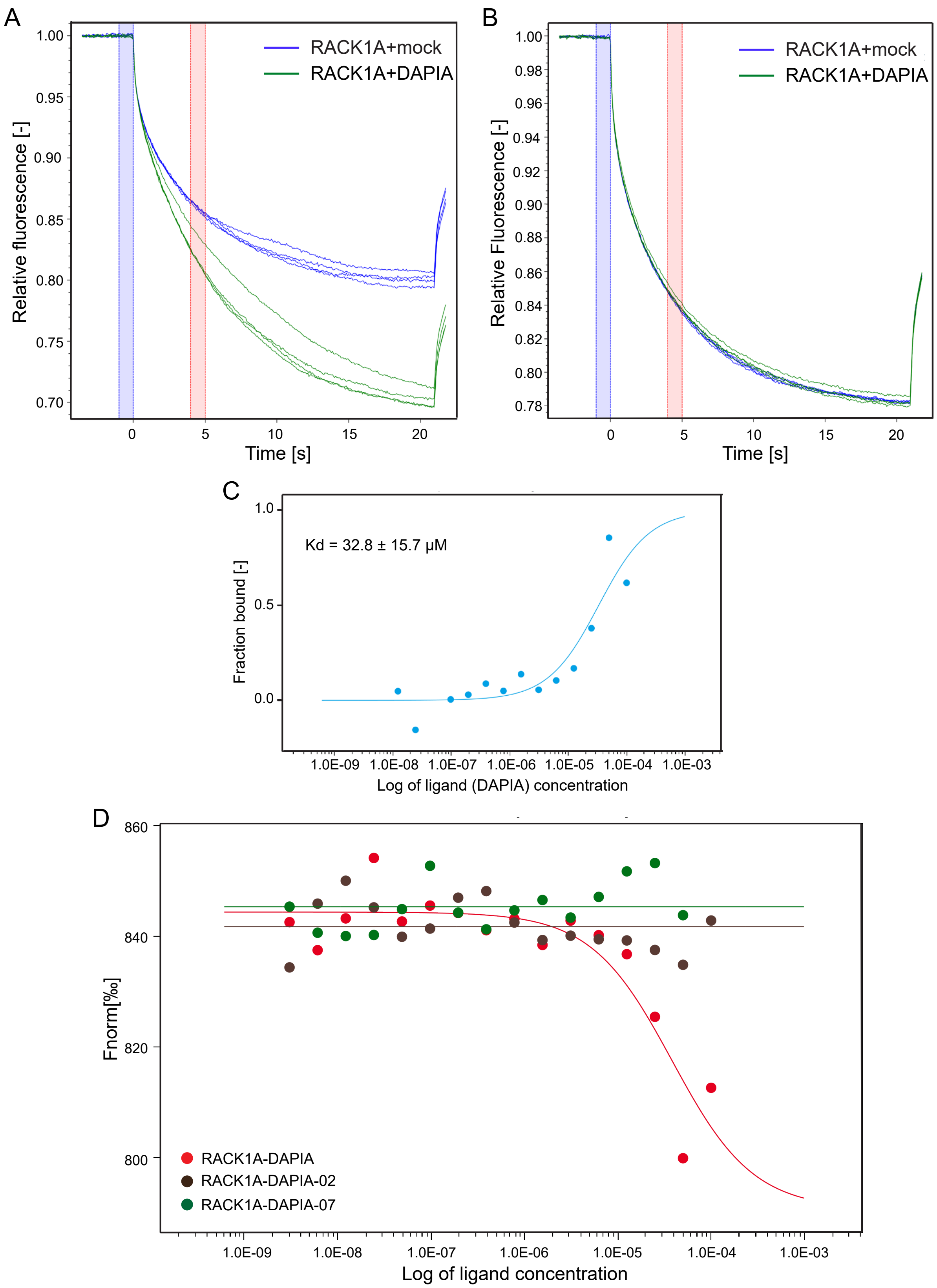


**Figure S4.** Direct binding between DAPIA and RACK1A validated by micro-scale thermophoresis (MST) analysis. (*A* and *B*) Qualitative verification of RACK1A and DAPIA binding in the absence (*A*) and presence (*B*) of Tween 20 or PIC. The signal to noise (S/N) value, calculated as the ratio of the response amplitude to the noise of the measurements (SD of the replicates) was 46.9 and 1.4 for DAPIA-treated samples in *A* and *B*, respectively. (*C*) MST analysis ligand dose-response curve; Kd = mean ± SD of four independent replicates. (*D*) Quantification of binding affinity between RACK1A and DAPIA or the inactive DAPIA analogs DAPIA-02 and DAPIA-07.

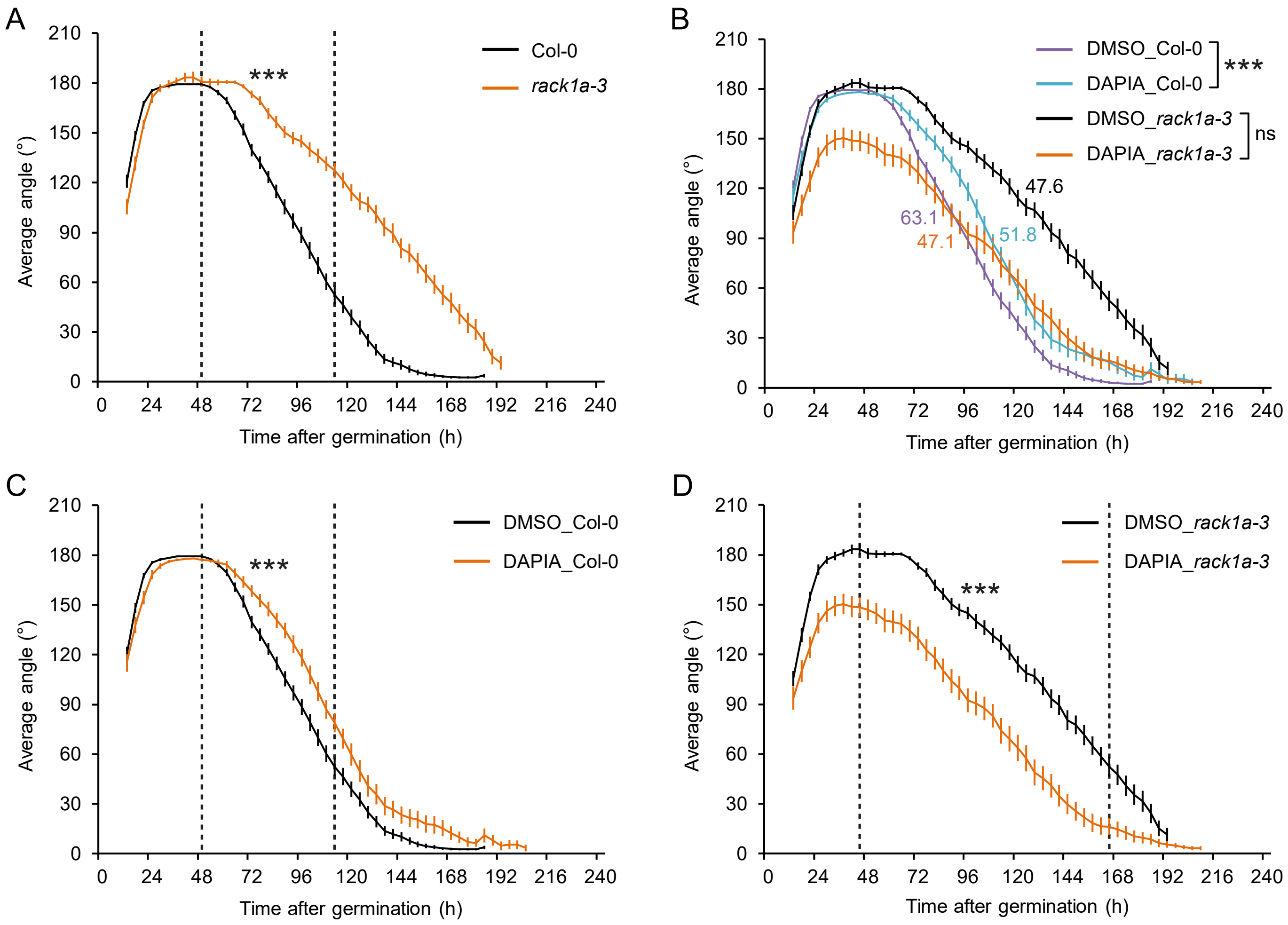


**Figure S5.** Apical hook development and effects of DAPIA treatment in *rack1a-3*. (*A* to *D*) Kinematics of apical hook angle in Col-0 and *rack1a-3*, as measured every 4 h for 10 d of growth starting from germination (0 h) in darkness on medium supplemented with DMSO (mock) or 10 µM DAPIA. Error bars represent SEM; *N* = 29-46 seedlings. Dashed lines in *A*, *C* and *D* indicate the late maintenance-opening phase (from the maximum mean hook angle to the first mean hook angle below 30% of the maximum) of the WT or mock-treated control, for which asterisks indicate significantly different kinematic curves (CGGC method; ***$P < 0.001$). Color-coded values beside the curves in *B* represent the rate of early hook opening, expressed as the mean slope angle in degrees of the late maintenance-opening phase, for which asterisks indicate significant differences (Wilcoxon rank sum test; ns – not significantly different; ***$P < 0.001$).

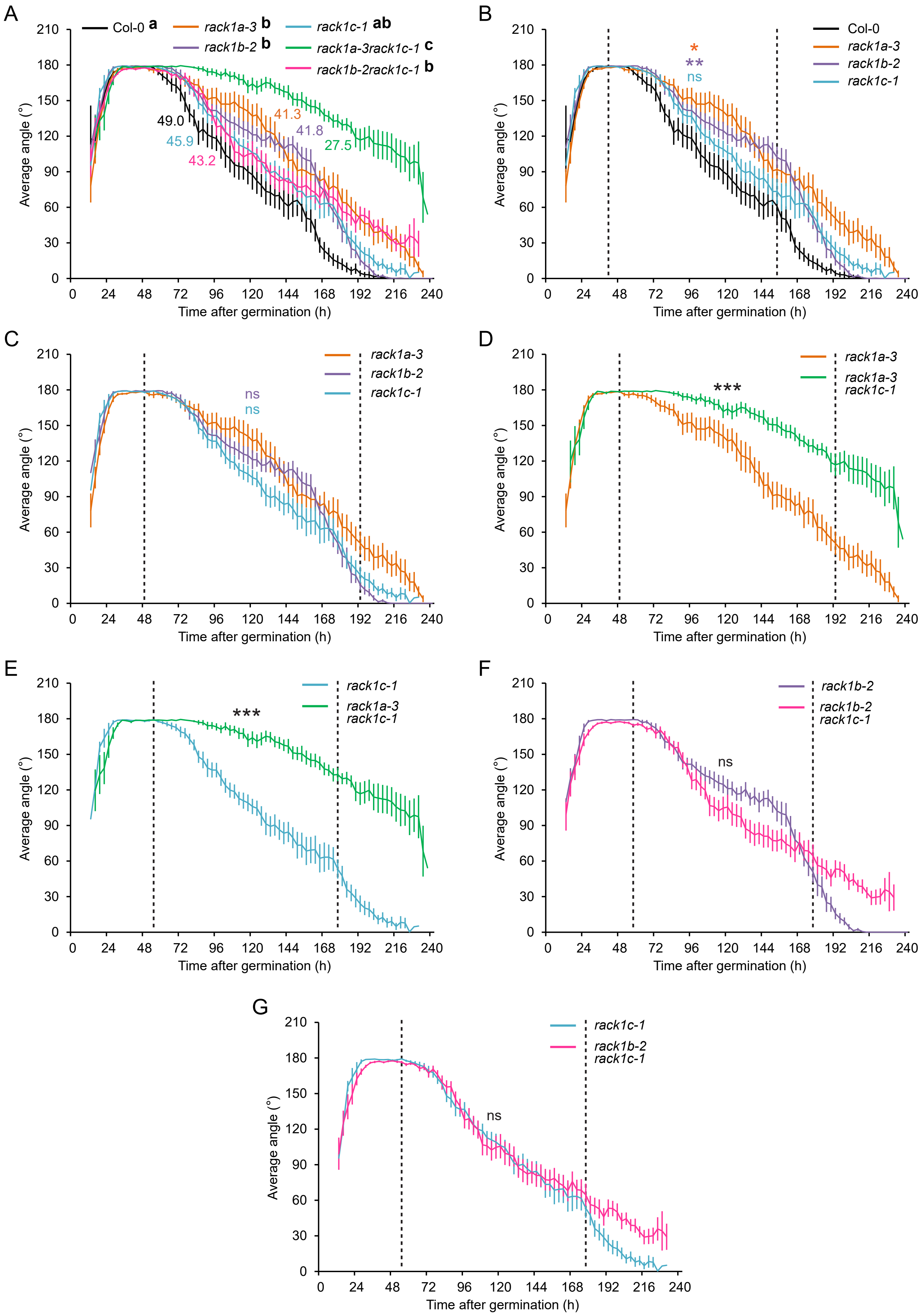
A
Col-0 a
rack1a-3 b
rack1b-2 b
rack1c-1 ab
rack1a-3rack1c-1 c
rack1b-2rack1c-1 b
49.0
45.9
43.2
41.3
41.8
27.5
B
Col-0
rack1a-3
rack1b-2
rack1c-1
*
**
ns
C
rack1a-3
rack1b-2
rack1c-1
ns
ns
D
rack1a-3
rack1a-3 rack1c-1
***
E
rack1c-1
rack1a-3 rack1c-1
***
F
rack1b-2
rack1b-2 rack1c-1
ns
G
rack1c-1
rack1b-2 rack1c-1
ns
Average angle (°)
Time after germination (h)

**Figure S6.** Apical hook development in single and double mutants of *RACK1A*, *B* and *C*. (*A* to *G*) Kinematics of apical hook angle in Col-0, *rack1a-3, rack1b-2, rack1c-1, rack1a-3rack1c-1* and *rack1b-2rack1c-1*, as measured every 3 h for 10 d of growth starting from germination (0 h) in darkness. Error bars represent SEM; *N* = 13-14 seedlings. Color-coded values beside the curves in *A* represent the rate of early hook opening, expressed as the mean slope angle in degrees of the late maintenance-opening phase (from the maximum mean hook angle to the first mean hook angle below 30% of the maximum), for which different letters beside the genotype names indicate significant differences at $P < 0.05$ (Wilcoxon rank sum test). Dashed lines in *B* to *G* indicate the late maintenance-opening phase of the "control" genotype (Col-0 in *B*, *rack1a-3* in *C* and *D*, *rack1c-1* in *E* and *G*, *rack1b-2* in *F*) to which the other genotypes were statistically compared and for which asterisks indicate significantly different kinematic curves (CGGC method; ns - not significantly different; *$P < 0.05$; **$P < 0.01$; ***$P < 0.001$). In *B* and *C*, statistical analyses are color-coded to indicate the relevant genotype to which Col-0 (*B*) and *rack1a-3* (*C*) were statistically compared.

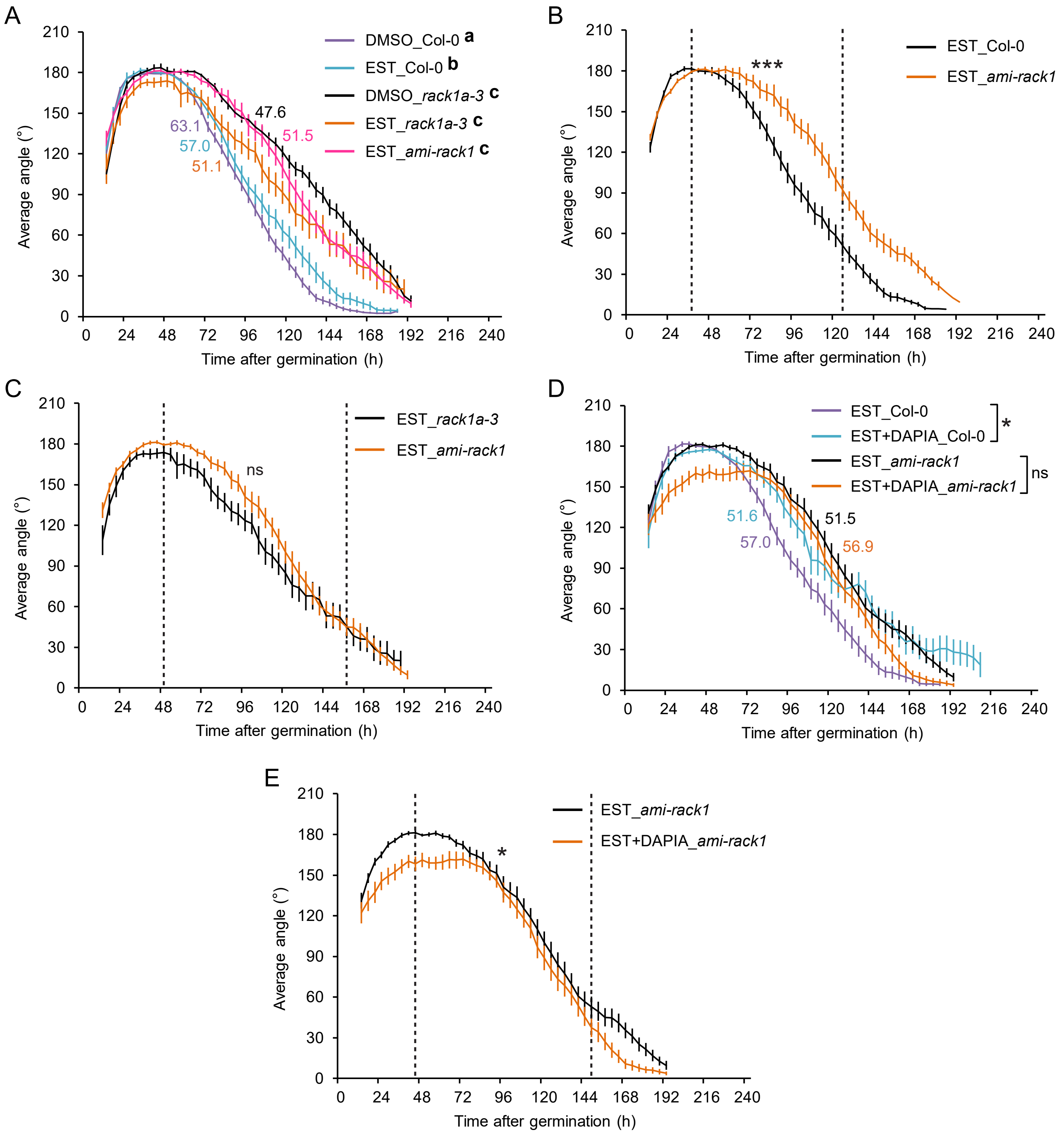


**Figure S7.** Apical hook development and effects of DAPIA treatment in single and inducible triple mutants of *RACK1A*, *B* and *C*. (*A* to *E*) Kinematics of apical hook angle in Col-0, *rack1a-3* and *amiR-rack1-es1* (*ami-rack1*), as measured every 4 h for 10 d of growth starting from germination (0 h) in darkness on medium supplemented with DMSO (mock) or 10 μM estradiol, DAPIA, or both. Error bars represent SEM; *N* = 14-46 seedlings. Color-coded values beside the curves in *A* and *D* represent the rate of early hook opening, expressed as the mean slope angle in degrees of the late maintenance-opening phase (from the maximum mean hook angle to the first mean hook angle below 30% of the maximum), for which different letters (*A*) or asterisks (*D*) beside the genotype names indicate significant differences at *P* < 0.05 (Wilcoxon rank sum test; ns – not significantly different). Dashed lines in *B*, *C* and *E* indicate the late maintenance-opening phase of the mock-treated control, for which asterisks indicate significantly different kinematic curves (CGGC method; ns - not significantly different; **P* < 0.05; ****P* < 0.001).

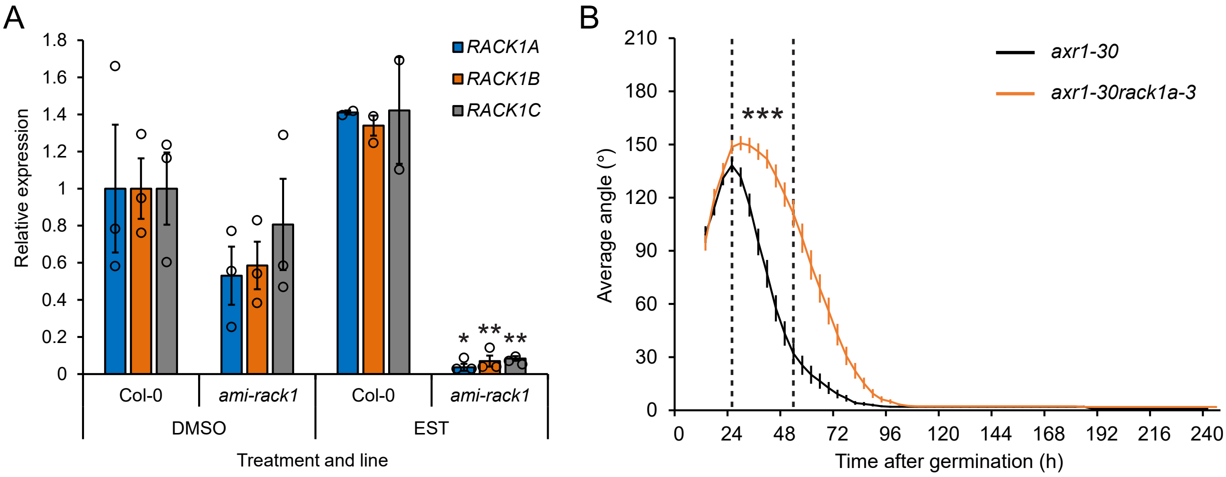


**Figure S8.** Estradiol treatment strongly decreases *RACK1A*, *B* and *C* expression in the *amiR-rack1-es1* mutant and the *rack1a-3* mutation partially rescues the hook opening phenotype in *axr1-30*. (*A*) Expression of *RACK1A*, *B* and *C* relative to the WT mock in the upper shoots of 4-day-old Col-0 and *amiR-rack1-es1* (*ami-rack1*) seedlings grown in darkness on medium supplemented with DMSO (mock) or 10 µM estradiol (EST). Error bars represent SEM and asterisks indicate significant differences compared to the WT mock (Student's T-test; **P* < 0.05; ***P* < 0.01); *N* = 3 biological replicates. (*B*) Kinematics of apical hook angle in *axr1-30* and combination of *axr1-30* with *rack1a-3*, as measured every 4 h for 10 d of growth starting from germination (0 h) in darkness. Error bars represent SEM; *N* = 35-41 seedlings. Dashed lines indicate the late maintenance-opening phase (from the maximum mean hook angle to the first mean hook angle below 30% of the maximum) of the *axr1-30* "control", for which asterisks indicate significantly different kinematic curves (CGGC method; ****P* < 0.001).

**Dataset S1.** Proteins enriched in DARTS assay identified by LC-MS/MS. (*A*) Summary of the identified proteins. RACK1A is highlighted in red. (*B* to *D*) Comparison_P100 (*B*), Comparison_P300 (*C*) and Comparison_P0 (*D*) show quantification results for samples subjected to proteolytic digestion by mixing with pronase at enzyme:protein substrate ratios of 1:100, 1:300 or 0 (no pronase), respectively.

## SI References


1. S. K. Hotton, R. A. Eigenheer, M. F. Castro, M. Bostick, J. Callis, AXR1-ECR1 and AXL1-ECR1 heterodimeric RUB-activating enzymes diverge in function in *Arabidopsis thaliana*. *Plant Mol. Biol.* 75, 515-526 (2011).
2. T. Ulmasov, J. Murfett, G. Hagen, T. J. Guilfoyle, Aux/IAA proteins repress expression of reporter genes containing natural and highly active synthetic auxin response elements. *Plant Cell* 9, 1963-1971 (1997).
3. G. Parry *et al*., Complex regulation of the TIR1/AFB family of auxin receptors. *Proc. Natl. Acad. Sci. U. S. A.* 106, 22540-22545 (2009).
4. P. Nagpal *et al*., *AXR2* encodes a member of the Aux/IAA protein family. *Plant Physiol.* 123, 563-574 (2000).
5. Q. Tian, J. W. Reed, Control of auxin-regulated root development by the *Arabidopsis thaliana SHY2/IAA3* gene. *Development* 126, 711-721 (1999).
6. Y. Okushima *et al*., Functional genomic analysis of the *AUXIN RESPONSE FACTOR* gene family members in *Arabidopsis thaliana*: unique and overlapping functions of *ARF7* and *ARF19*. *Plant Cell* 17, 444-463 (2005).
7. B. Orosa-Puente *et al.*, Root branching toward water involves posttranslational modification of transcription factor ARF7. *Science* 362, 1407-1410 (2018).
8. S. K. Powers *et al*., Nucleo-cytoplasmic partitioning of ARF proteins controls auxin responses in *Arabidopsis thaliana*. *Mol. Cell* 76, 177-190 (2019).
9. Z. Cheng *et al*., Pathogen-secreted proteases activate a novel plant immune pathway. *Nature* 521, 213-216 (2015).
10. J. Guo, J.-G. Chen, *RACK1* genes regulate plant development with unequal genetic redundancy in *Arabidopsis*. *BMC Plant Biol.* 8, 108 (2008).
11. J. Guo et al., RACK1 is a negative regulator of ABA responses in *Arabidopsis*. *J. Exp. Bot.* 60, 3819-3833 (2009).
12. C.-Y. Liao *et al*., Reporters for sensitive and quantitative measurement of auxin response. *Nat. Methods* 12, 207-210 (2015).
13. K. Edwards, C. Johnstone, C. Thompson, A simple and rapid method for the preparation of plant genomic DNA for PCR analysis. *Nucleic Acids Res.* 19, 1349 (1991).
14. G. W. Haughn, C. Somerville, Sulfonylurea resistant mutants of *Arabidopsis thaliana*. *Mol. Genet. Genomics* 204, 430-434 (1986).
15. T. Vain *et al*., Selective auxin agonists induce specific AUX/IAA protein degradation to modulate plant development. *Proc. Natl. Acad. Sci. U. S. A.* 116, 6463-6472 (2019).
16. H. Xu, C. Wolf, Copper catalyzed coupling of aryl chlorides, bromides and iodides with amines and amides. *Chem. Commun.* 13, 1715-1717 (2009).
17. L. A. Mevellec *et al*., Substituted 4,5,6,7-tetrahydro-pyrazolo[1,5-a]pyrazine derivatives and 5,6,7,8-tetrahydro-4H-pyrazolo[1,5-a][1,4]diazepine derivatives as ROS1 inhibitors. Patent no. WO 2015/144799 A1 (2015).
18. S. Ninkovic, *et al*., 6 substituted 2-heterocyclylamino pyrazine compounds as CHK-1 inhibitors. Patent no. WO 2010/016005 A1 (2010).
19. C. Béziat, J. Kleine-Vehn, E. Feraru, Histochemical staining of β-glucuronidase and its spatial quantification. *Methods Mol. Biol.* 1497, 73-80 (2017).
20. B. Pařízková *et al*., New fluorescent auxin probes visualise tissue-specific and subcellular distributions of auxin in Arabidopsis. *New Phytol.* 230, 535-549 (2021).
21. B. Lomenick, G. Jung, J. A. Wohlschlegel, J. Huang, Target identification using drug affinity responsive target stability (DARTS). *Curr. Protoc. Chem. Biol.* 3, 163-180 (2011).
22. I.-F. Chang, K. Szick-Miranda, S. Pan, J. Bailey-Serres, Proteomic characterization of evolutionarily conserved and variable proteins of Arabidopsis cytosolic ribosomes. *Plant Physiol.* 137, 848-862 (2005).
23. S. Forli *et al*., Computational protein–ligand docking and virtual drug screening with the AutoDock suite. *Nat. Protoc.* 11, 905-919 (2016).

24. G. M. Morris *et al*., AutoDock4 and AutoDockTools4: automated docking with selective receptor flexibility. *J. Comput. Chem.* 30, 2785-2791 (2009).
25. E. F. Pettersen *et al*., UCSF Chimera -- a visualization system for exploratory research and analysis. *J. Comput. Chem.* 25, 1605-1612 (2004).
26. S. M. Doyle *et al.*, A role for the auxin precursor anthranilic acid in root gravitropism via regulation of PIN-FORMED protein polarity and relocalisation in *Arabidopsis*. *New Phyt.* 223, 1420-1432 (2019).
27. J. Vandesompele *et al.*, Accurate normalization of real-time quantitative RT-PCR data by geometric averaging of multiple internal control genes. *Genome Biol.* 3:RESEARCH0034 (2002).
28. C. M. Elso *et al*., Leishmaniasis host response loci (lmr13) modify disease severity through a Th1/Th2-independent pathway. *Genes Immun.* 5, 93-100 (2004).
